# Numerical operations in living cells by programmable RNA devices

**DOI:** 10.1126/sciadv.aax0835

**Published:** 2019-08-21

**Authors:** Kei Endo, Karin Hayashi, Hirohide Saito

**Affiliations:** 1 Department of Life Science Frontiers, Center for iPS Cell Research and Application, Kyoto University, 53 Kawahara-cho, Shogoin, Sakyo-ku, Kyoto 606-8507, Japan.; 2 Department of Computational Biology and Medical Sciences, Graduate School of Frontier Sciences, The University of Tokyo, 5-1-5 Kashiwanoha, Kashiwa-shi, Chiba 277-8562, Japan.

## Abstract

Integrated bioengineering systems can make executable decisions according to the cell state. To sense the state, multiple biomarkers are detected and processed via logic gates with synthetic biological devices. However, numerical operations have not been achieved. Here, we show a design principle for messenger RNA (mRNA) devices that recapitulates intracellular information by multivariate calculations in single living cells. On the basis of this principle and the collected profiles of multiple microRNA activities, we demonstrate that rationally programmed mRNA sets classify living human cells and track their change during differentiation. Our mRNA devices automatically perform multivariate calculation and function as a decision-maker in response to dynamic intracellular changes in living cells.

## Teaser

Programmable RNA devices recapitulate intracellular information by multivariate linear combinations in living cells.

## INTRODUCTION

To engineer living cells and organisms, artificial systems that function in response to cellular states have been programmed using synthetic devices made of biomolecules (*1*–*3*). Switches (*4*–*10*), sometimes with inverters (*11*, *12*), and split systems (*13*) sense a biomarker to change their activity, transmitting cellular information to the device. Logic gates (*11*, *14*–*17*) process multiple inputs of biomarkers, extending the decision-making capacity. The synthesis of a more intelligent system that functions according to complex criteria requires the numerical operation of numerous biomarkers. However, molecular devices that perform multivariate numerical operations in living cells remain undeveloped. Moreover, the number of applicable devices for sensing, signal transmitting, and decision-making limits the number of accessible biomarkers.

**Fig. 1:**
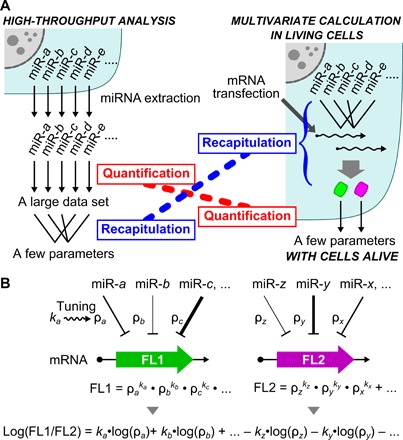
Scheme for a multivariate linear combination in living cells. (**A**) Two steps in the calculation of an intracellular miRNA profile. The left panel shows a canonical approach, such as high-throughput analysis, in which the detection of a large dataset for miRNA molecules in a cell is followed by multivariate statistics to recapitulate the profile with few parameters. The right panel shows our approach, in which the profile is recapitulated with an mRNA device before signal detection. (**B**) In our device, mRNA responds to multiple miRNAs independently and with high sensitivity to output the coded fluorescent protein (FL1 or FL2). The values of ρ denote miRNA activity that represses the reporter protein expression and are tuned by specific tuning factors, *k*. A linear combination of multiple miRNA activities in a cell is obtained as the ratio of two fluorescent reporters.

mRNA-based technologies, i.e., artificial riboswitches, have attracted attention in device construction due to their ability to respond to molecules of interest and to encode genes of interest in a single molecule. Synthetic mRNAs do not integrate into the genome of host cells and rapidly decrease in cells (half-life of approximately 8 hours), offering safe medical application, such as cellular therapeutics (*18*). In addition, mRNA devices can respond to a target microRNA (miRNA) that functions as a biomarker, controlling protein production and cell function (*19*, *20*). miRNA profiles are usually determined by quantitative measurements from high-throughput analysis (*21*), followed by multivariate statistics to obtain new parameters that recapitulate the cellular state (Fig. 1A, left) (*22*). Using the resulting parameters, cells of interest have been identified and classified; however, by this time, the cells have already been lysed in the experiments. The detection of miRNAs in living cells with commonly used probes, which function in a one-to-one correspondence (*23*), limits the number of used miRNAs because of limits in the simultaneous detection of multiple signals, making it difficult to perform numerical operations in live cells. The design of a device that simultaneously and quantitatively detects multiple signals (one-to-many device; Fig. 1A, right) requires rational means for integrating the response to multiple miRNAs and for tuning the sensitivity to miRNA activity independently (Fig. 1B).

In this study, we show synthetic mRNA devices that operate multivariate numerical operations in live cells and recapitulate intracellular information. To use a quantitative profile of multiple miRNAs, we developed a design principle for programmable mRNA devices. A single mRNA detects multiple miRNA activities in a quantitatively additive manner and in an independently tunable manner. On the basis of these two principles, we succeeded in rationally designing mRNA devices to classify living human cells and to track dynamic states during cellular differentiation according to miRNA activity.

## RESULTS

### Design principle for mRNA devices that perform multivariate calculations

**Fig. 2:**
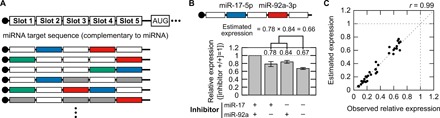
Quantitatively additive detection of miRNA activity by a synthetic five-slot mRNA. (**A**) The design of a synthetic mRNA that contains five slots for miRNA target sequences complementary to the miRNAs in the 5′UTR. The bottom part shows five-slot mRNAs responding to two or three miRNAs. Colored boxes indicate occupation of the slots by a target sequence as follows: gray, miR-34-a-5p; blue, miR-17-5p; red, miR-92a-3p; and green, miR-21-5p. Blank boxes depict empty slots, which are sequences of the same length as the target sequence and free from an miRNA target sequence. (**B**) An example result of a five-slot mRNA that responds to miR-17-5p and miR-92a-3p in HeLa cells. The design of the slots is shown above. Relative expressions are defined as the reporter expression normalized by the expression in the presence of miRNA inhibitors to both miRNAs (+/+). Values are presented above the bars. Error bars indicate the mean ± SD (*n* = 3). Calculation of the estimated expression is depicted above the chart. (**C**) Comparison of the relative expression with the estimated expression. A dot in the plot indicates the result of a five-slot mRNA responding to two or three miRNAs. Three independent experiments of 12 mRNAs are shown. Correlation coefficient (*r*) = 0.99 (for the means).

First, we designed synthetic mRNAs that respond to multiple miRNA inputs and examined their behavior. The activity of an miRNA in a living cell can be efficiently detected by an mRNA that contains a completely complementary sequence to the target miRNA in the 5′ untranslated region (5′UTR) and expresses a reporter fluorescent protein (*19*, *20*). Notably, our previous study indicated that the transfection of a reporter mRNA with a completely complementary sequence to an miRNA does not affect the endogenous miRNA expression level or cell state (*19*). Therefore, we added slots for the miRNA target sequence in the 5′UTR and synthesized a series of five-slot mRNAs that respond to two or three distinct miRNAs (Fig. 2A). We used HeLa cells and chose miR-34a-5p, miR-17-5p and miR-92a-3p, and miR-21-5p as proof-of-principle experiments, since their activities are weak, intermediate, and strong in these cells, respectively, according to our previous study (*20*). Synthesized five-slot mRNAs expressing humanized monomeric Azami-Green 1 (hmAG1) were transfected into HeLa cells together with tag blue fluorescence protein (tagBFP) mRNA as a transfection control and with inhibitors of the corresponding miRNAs. The expression of hmAG1 relative to tagBFP was measured by flow cytometry 24 hours after the transfection. Figure 2B shows the case of a five-slot mRNA responding to miR-17-5p at slot 2 and to miR-92a-3p at slot 4. The reporter expression in the presence of both miRNA inhibitors indicated the mRNA expression without any miRNA effect and was used as the standard (see Supplementary Text for the definition of the indices). Relative expressions in the presence of either miRNA inhibitor (0.78 and 0.84 in Fig. 2B) revealed the activity of the other miRNA in a condition in which no off-target effect of the miRNA inhibitors was observed (fig. S1). In the absence of both inhibitors, the relative expression under the control of both miRNAs (0.67) was close to the estimated expression (0.78 × 0.84 = 0.66), which is the product of the expression in the presence of either miRNA inhibitor (miR-17 or miR-92a). To confirm the generality of this finding, we randomly constructed 12 five-slot mRNAs (Fig. 2A and table S1) and compared the estimated expression with the relative expression observed in the absence of the corresponding miRNA inhibitors. These two values showed high consistency (correlation coefficient, *r* = 0.99; Fig. 2C and Table 1), indicating that a synthetic mRNA with multiple slots detects the activities of multiple miRNAs in a quantitatively additive manner.

**Table 1:** Summary of the model accuracy.

**Model**	**Figure**	**Determination** **coefficient (*R*^2^)**	**Root mean** **square error**
Additivity	Fig. 2C	0.99	0.029
Positional effect (overall)	Fig. 3B	0.96	0.066
miR-34a-5p		0.75	0.044
miR-17-5p		0.92	0.067
miR-21-5p		0.92	0.071
miR-92a-3p		0.91	0.071
Distance dependency (overall)	Fig. 3C	0.95	0.053
miR-34a-5p		0.96	0.014
miR-17-5p		0.70	0.072
miR-21-5p		0.97	0.045
miR-92a-3p		0.99	0.062

**Fig. 3:**
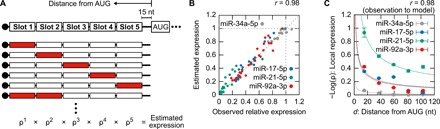
Independently tunable detection of miRNA activity by a synthetic five-slot mRNA. (**A**) A series of five-slot mRNAs that contain one to three copies of the identical miRNA target sequences. A part of the series responding to miR-92a-3p (red boxes) is illustrated. The 5′UTR sequences are shown in table S1. The equation below represents the estimated expression, which is the product of the relative expressions repressed by the miRNA at each slot (ρ). (**B**) Comparison of the relative expression with the estimated expression. A dot in the plot indicates the result of a five-slot mRNA responding to the indicated miRNA with one slot or more. The values are the mean of three independent analyses. *r* = 0.98 (for all dots). (**C**) Relationship between the distance of a slot from the start codon (*d*) and the repression by the indicated miRNAs at the slot (local repression). Error bars indicate the mean ± SD (*n* = 3). Dotted lines are curves of the exponential model with the global constant = −0.56 (eqs. S5 and S6 in Supplementary Text). *r* = 0.98 (for all dots). nt, nucleotides.

We found that the effect of the miRNA depended on the slot position of its target sequence in the mRNAs. We therefore investigated the positional effect of miRNA target sequences in the 5′UTR of synthetic mRNAs. We designed a five-slot mRNA, one to three slots of which are occupied by the identical miRNA target sequence (Fig. 3A and table S1). We synthesized a series of the five-slot mRNAs for the four model miRNAs and determined the relative expression in the absence of the corresponding miRNA inhibitor to that in its presence. According to our findings in Fig. 2C, the relative expression is a product of the relative expressions repressed by the miRNA at each slot (denoted as ρ in Fig. 3A). Thus, we estimated ρ values of each miRNA at each slot by fitting the dataset of the relative expressions of the four series. In all cases, calculated ρ values well explained the behavior of the five-slot mRNAs (*r* = 0.98; Fig. 3B and Table 1). Notably, ρ values decreased as the position number increased (fig. S2), and the relative expressions of single-slot mRNAs, the slot of which is located approximately 20 nucleotides from both the 5′ end and the start codon, were close to ρ values at slot 5 (fig. S2). These results suggest that the positional effect of the slot depends on the distance of the slot from the start codon (denoted as *d*). To investigate the relationship between the distance and the sensitivity of the slots independent of the miRNA species, we simultaneously fitted ρ values from the four model miRNAs with an exponential model (Fig. 3C and Table 1). As a result, local repression, which we defined as −log(ρ*^i^*), was well described as the product of miRNA-specific repression, −log(ρ), and a tuning factor, *k* = *d*^−0.56^ (eqs. S5 and S6 in Supplementary Text). This finding shows that moving the position of the target sequence in the 5′UTR can independently and predictively tune the synthetic mRNA sensitivity to the miRNA of interest.

Combination of the two principles (additivity and tunability) enables recapitulation of an miRNA activity profile in a living cell by multivariate linear combinations. The activities of multiple miRNAs in a cell are quantitatively summed up and detected by a synthetic mRNA with multiple slots (Fig. 2C). Besides, sensitivity for miRNA activity is independently tunable by the distance of the slot from the start codon (Fig. 3C). Thus, the expression of a reporter fluorescent protein from a multislot synthetic mRNA represents a linear combination of miRNA activities (Fig. 1B; see also eq. S7 in Supplementary Text). In this model, coefficients for multiple miRNA activities (tuning factor, *k* = *d*^−0.56^) must have positive values. The fluorescence ratio of two multislot mRNAs expands the range of the coefficients to negative values (−*k* = −*d*^−0.56^; Fig. 1B and eq. S8 in Supplementary Text) and improves the resolution for cell identification (*20*), that is, two synthetic multislot mRNAs enable the numerical operations of miRNA activities in living cells as the fluorescence ratio.

### Design of mRNA devices to classify living cells

**Fig. 4:**
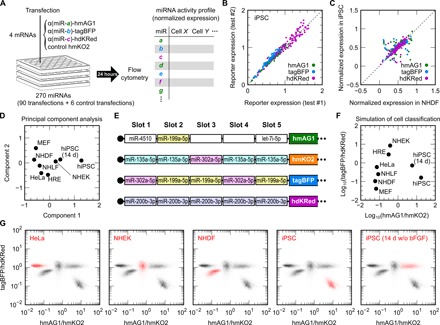
Classification of living cells by a set of five-slot mRNAs based on miRNA activity profiles. (**A**) Schematic illustration of the miRNA activity screening. A set of three single-slot mRNAs that respond to distinct miRNAs (miR-*a*, miR-*b*, and miR-*c*) and encode hmAG1, tagBFP, and hdKRed, respectively, and control hmKO2 mRNA were transfected into target cultured cells on a 24-well plate. The cells were analyzed by flow cytometry 24 hours later. The right table depicts miRNA activity profiles in analyzed cell types, which are determined on the basis of the expressions of the single-slot reporter mRNAs. Four plates (96 transfections) covered the screening of 270 miRNAs, as well as control transfections. (**B**) Obtained reporter expressions in hiPSCs from two independent screenings. Green, blue, and purple dots indicate single-slot mRNAs encoding hmAG1, tagBFP, and hdKRed, respectively. (**C**) Comparison of normalized miRNA activity profiles in NHDFs and hiPSCs. The normalized expressions are the mean of two screenings. See Supplementary Text and fig. S3 for details. (**D**) Classification of eight cell types by PCA of the 270 miRNAs. The cell types are plotted on the first two components (components 1 and 2). (**E**) The design of a set of four five-slot mRNAs that maximizes the variance of the eight cell types. Slots are occupied by the target sequence to the indicated miRNAs. Blank boxes denote empty slots. (**F**) Simulated classification of the eight cell types with the transfection of the mRNA set shown in (E). Axes are the fluorescence ratios of hmAG1/hmKO2 and tagBFP/hdKRed. (**G**) Live cell classification with the set of the four synthetic mRNAs and flow cytometry. Merged density plots of the transfected cells on the two fluorescence ratios are shown. The indicated cells are presented in red density, other cells in black density.

To prove our concept, we attempted to classify eight different living cell types by two synthetic parameters calculated from the activities of multiple miRNAs using a set of four five-slot mRNAs. Although profiles of miRNA expression have been analyzed in a high-throughput manner, only one-third of the expressed miRNAs were reported to show activity that represses protein production from reporter mRNAs (*24*). Thus, the profiles of miRNA activities in human cells were determined via a screening with single-slot mRNAs (Fig. 4A). From the 1320 human miRNAs deposited in the database miRBase (release 19) (*25*), we excluded the 293 miRNAs that contain CAU, as this complementary sequence causes an upstream start codon in the mRNAs. To screen diverse miRNA sequences, we also excluded miRNAs with high sequence similarity. Then, we selected 270 miRNAs according to their expression level (read count in the database) and prepared a library of 270 mRNAs that respond to these miRNAs (table S2). In the library, one-third of single-slot mRNAs express hmAG1, another third tagBFP, and the final third humanized dimeric Keima-Red (hdKRed). We mixed the three mRNAs and humanized monomeric Kusabira-Orange 2 (hmKO2) mRNA (transfection control) and transfected sets of the four mRNAs into different human primary cultured cells: normal human dermal fibroblasts (NHDFs), normal human lung fibroblasts (NHLFs), neonatal normal human epithelial keratinocytes (NHEKs), and human renal epithelial cells (HREs), as well as NHDF-derived, human induced pluripotent stem cells (hiPSCs), HeLa cells, and murine embryonic fibroblasts (MEFs). We also analyzed spontaneously differentiated hiPSCs cultured for 14 days without basic fibroblast growth factor (bFGF) (*26*). Notably, we used the same lot of four mRNA mixtures throughout the screening to avoid noises in the miRNA activity detection caused by the mRNA mixing. We subjected the transfected cells to flow cytometry after 24 hours to determine the activity profiles of the 270 miRNAs. The results from two independent screenings using hiPSCs showed high consistency (Fig. 4B), indicating validity of our screening. Biases caused by differences among cell types and among reporter fluorescent proteins were normalized (Fig. 4C and fig. S3) before principal component analysis (PCA), which is based on a multivariate linear combination (Fig. 4D). To measure similar synthetic parameters with the principal components for cell classification directly from living cells, we designed a set of four five-slot mRNAs that maximizes variance among the eight analyzed cell types (Fig. 4E). With the designed mRNA set, the cells were classified according to two fluorescence ratios: hmAG1/hmKO2 and tagBFP/hdKRed (Fig. 4F). The transfection of the mRNA set experimentally separated these cell types in a two-dimensional (2D) plane for the identification and isolation of living cells (Fig. 4G). Among the shown five cell types, HeLa cells, iPSCs, and differentiated hiPSCs formed densities at almost the same position as in the simulation (Fig. 4F). As for NHEK and NHDF, their density positions on the *x* axis (hmAG1/hmKO2) were as expected, but those on the *y* axis (tagBFP/hdKRed) were closer to the center than expected. The control mRNA set and a single-slot mRNA set failed to separate these cell types (fig. S4, A and B).

**Fig. 5:**
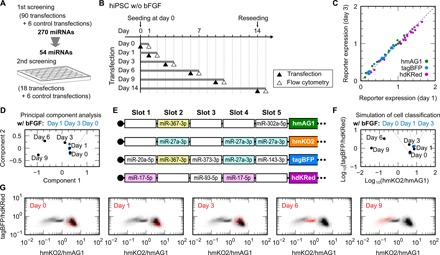
Tracking changes in hiPSCs by a set of five-slot mRNAs based on miRNA activity profiles. (**A**) Schematic illustration of the second screening. According to differences between hiPSCs and differentiated hiPSCs revealed by the first screening, 54 of 270 miRNAs were selected for the second screening. Together with control transfections, 54 single-slot mRNAs made 24 sets of mRNA transfection. (**B**) Time course for tracking changes in hiPSCs. hiPSCs were cultured without bFGF for up to 14 days. The cells were transfected with 24 sets of mRNAs at the indicated days (filled triangles) and, 24 hours later, analyzed by flow cytometry (open triangles). Before the transfection at day 14, the cells were reseeded on new culture plates. (**C**) Reporter expressions between days 1 and 3. Green, blue, and purple dots denote single-slot mRNAs encoding hmAG1, tagBFP, and hdKRed, respectively. (**D**) Classification of hiPSCs in the time course by PCA of the 54 miRNAs. For comparison, hiPSCs cultured with bFGF for the indicated days were also subjected to the second screening and the analysis (blue dots). The cells are plotted on the first two components. (**E**) The design of a set of four five-slot mRNAs that maximizes the variance of the hiPSCs. (**F**) Simulated classification of the hiPSCs using the set shown in (E). (**G**) Density plots of the transfected hiPSCs. Plots are shown as in Fig. 4G except that the *x* axis represents hmKO2/hmAG1.

Last, we tracked changes in the miRNA activity profile of differentiating hiPSCs with another set of synthetic mRNAs. According to the first screening (Fig. 4A), we selected 54 miRNAs that were differential between hiPSCs and differentiated hiPSCs and used them for the second screening (Fig. 5A). In the second screening, we cultured hiPSCs without bFGF and transfected them with single-slot mRNA sets on days 0, 1, 3, 6, 9, and 14 (Fig. 5B). Among the cells at the tested time points, we hardly observed a bias in the fluorescence ratios (Fig. 5C) and thus used the ratios to perform PCA (Fig. 5D) and to design another set of four five-slot mRNAs that maximizes the variance of the cells during differentiation (Fig. 5, E and F). Consequently, this mRNA set outputs two synthetic parameters calculated from the activities of eight miRNAs (Fig. 5E). According to these parameters, the transfection of the mRNA set made a trajectory of the states of hiPSCs during differentiation, which were still alive after the analysis (Fig. 5G). Consistent with the simulation (Fig. 5F), the designed mRNA set separated hiPSCs at different time points in the *x* axis direction, but only slightly in the *y*-axis direction. Among the cells, the density position of hiPSCs at day 6 was closer to the center than expected, particularly in the *y*-axis direction. Consistent with our design, the mRNA set for tracking hiPSCs distinguished the human primary cultures less effectively (fig. S4C), but expanded the separation space of the hiPSCs compared with that of human primary cultures (fig. S5). These results indicate that an mRNA set recapitulates the miRNA activity profiles of living cells in a manner specific to its design and performs numerical operations to distinguish the cell state.

## DISCUSSION

In this study, we rationally designed sets of four synthetic mRNAs that each respond to multiple miRNAs and regulate the expression of fluorescent proteins. The ratios of the fluorescent proteins produced from distinct mRNAs represent linear combinations of miRNA activities in a living cell. According to these synthetic parameters, the cells were separated in a 2D plane for isolation. Despite the limit in the number of used miRNAs and range of tuning factors, the predicted spaces for cell separation using a specific set of four mRNAs (Figs. 4F and 5F) were comparable to ideal spaces derived from 270 miRNAs (Figs. 4D and 5D). The transfected living cells separated in the 2D plane as expected; however, some cells diverged from the simulated positions, particularly along the *y* axis, implying poor accuracy in the calculation. One problem in our approach is the brightness of the reporter proteins. Relatively dark reporter proteins, such as hdKRed, require many mRNAs to be transfected, which, in turn, might affect the sensitivity of the miRNA activity measurement. Another problem is differences in the 5′UTR sequence. Although our devices work in principle, the insertion of different sequences may change the secondary structures and thus affect the rate of translation, as well as miRNA binding to the mRNAs. Further modulation in the mRNA design to reduce these noises (observed in Fig. 3B), including optimization of the fluorescent proteins and RNA secondary structures, should improve the calculation accuracy of the miRNA activity profiles.

The resolution and size of the cell separation space depend on the efficiency of the mRNA delivery. To separate the cells according to intracellular information at high resolution, the proportion of each mRNA device should be constant even if the total amount of mRNA delivered into each cell varies (*20*). To increase the dimension of the cell separation space, two additional mRNAs encoding other fluorescent proteins need to be delivered at a level sufficient for detection. As long as the means for the mRNA delivery meets these requirements, the means for the delivery of our devices is not limited to the lipofection shown in this study. Recently, materials for efficient mRNA delivery, such as dendrimers (*27*), gold nanoparticles (*28*), polyplex particles (*29*), and nanoparticles made of mRNA (*30*), have been developed. The use of these materials could enhance the capability of our devices to separate cells. Alternatively, our devices could be encoded into the genome to ensure their uptake by the cells (*31*). In this case, the devices continuously detect changes in the cell during a certain process (e.g., differentiation) rather than taking a snapshot of the moment.

On the basis of two principles, additivity and tunability, we could design a pair of mRNAs to output a new quantitative parameter that describes the activity of multiple miRNAs in single living cells. So far, synthetic devices detect and process multiple intracellular molecules only logically (*11*, *14*–*17*, *32*) and fail to provide quantitative information. Recently, one group reported an antagonistic and synergistic repression model to predict the expression of multislot mRNAs that include repeated miRNA-target sequences predominantly designed in the 3′UTR and transcribed in cells (*33*). The engineering of the 5′UTR limits the number of slots for the miRNAs, because the average 5′UTR length ranges between 100 and 200 nucleotides (*34*) and long 5′UTR lengths decrease the efficiency of the protein production (*35*). However, to our knowledge, our study is the first to achieve numerical operations in living cells comparably with the PCA obtained from lysed cells to quantitatively describe intracellular information. Our approach, in which multiple inputs are numerically processed, enables the identification and isolation of cells of interest with more complex criteria than ever for medical applications, such as cellular therapeutics and pharmacological screenings.

## MATERIALS AND METHODS

### Cell and culture

HeLa cells (American Type Culture Collection) were cultured in Dulbecco’s modified Eagle’s medium supplemented with 10% fetal bovine serum (FBS) and 1% antibiotic antimycotic solution (Sigma-Aldrich). Human primary cultured cells were expanded in the provided medium according to the manufacturer’s instructions. NHDFs (Lonza) and NHLFs (Lonza) were cultured with FBM-2 BulletKit containing 2% FBS (Lonza), neonatal NHEKs (Lonza) with KGM-Gold BulletKit (Lonza), and HREs (Lonza) with REGM BulletKit containing 0.5% serum (Lonza). MEFs (Lonza) were expanded in the same medium as HeLa cells. These primary cultured cells were used within 10 passages. hiPSCs derived from NHDFs (201B7) (*36*) were maintained with StemFit AK03N (AHS) on nippi iMatrix-511–coated well plates. Upon spontaneous differentiation (*26*), 3000 hiPSCs were seeded on iMatrix-511–coated six-well plates and cultured in StemFit AK03N without bFGF for 14 days. Cell culture without bFGF for 1, 3, and 6 or 9 days was started with 50,000, 10,000, and 3000 hiPSCs, respectively, on 24-well plates. The medium for hiPSCs was changed every second day.

### Sequence of synthetic mRNAs

The 5′UTR sequences of the synthetic mRNAs used in this study are available in tables S1 (for five-slot mRNAs) and S2 (for single-slot mRNAs). The mRNAs encode hmAG1 (Amalgaam), hmKO2 (Amalgaam), tagBFP (Evrogen), or hdKRed (Amalgaam) and are followed by a 3′UTR derived from *Mus musculus* α-globin mRNA and a 120-nucleotide poly-A tail (*19*, *20*, *37*). The 5′UTR sequence of the control mRNAs was 57 nucleotides long and free of an miRNA target sequence (*19*, *20*).

### Synthesis and transfection of mRNAs

The synthesis and transfection of reporter mRNAs were performed as described previously (*20*). Before polymerase chain reaction (PCR) amplification of the templates for the in vitro transcription (IVT), double-stranded DNA fragments of the open reading frame of several fluorescent proteins (hmAG1, hmKO2, tagBFP, and hdKRed) and of the negative control 5′UTR and the 3′UTR were prepared via PCR with specific primers from plasmids and single-stranded oligo DNAs (table S3). Then, the fragments were mixed and amplified via fusion PCR with T7Fwd5UTR and Rev120A containing T7 promoter and poly-T tract, respectively (table S3). To synthesize the IVT templates for miRNA-responsive mRNAs, one, two, or three single-stranded oligo DNAs for the designed 5′UTRs (tables S1 and S2) were mixed into the fusion PCR instead of the negative control 5′UTR fragment. From the purified IVT templates, mRNAs were synthesized by T7 RNA polymerase (MEGAscript T7 kit, Ambion) and capped cotranscriptionally in the presence of an antireverse cap analog (New England BioLabs). In the transcription reaction, all cytidine and uridine triphosphates were replaced by 5-methylcytidine and pseudouridine triphosphates (TriLink BioTechnologies), respectively. The transcripts were purified and then treated with Antarctic Phosphatase (New England BioLabs) to remove the phosphates in the 5′ terminus. The synthesized mRNAs were further purified with RNeasy MinElute Cleanup Kit (Qiagen) and stored at −20°C until use. The mRNAs (up to 500 ng) and mirVana miRNA inhibitors (up to 6 pmol; Applied Biosystems) were transfected into the cultured cells on 24-well plates via lipofection with 1 μl of StemFect (Stemgent) according to the manufacturer’s instructions. In the first screening of 270 single-slot mRNAs and following live cell classification, the transfections were performed according to a reverse transfection protocol with 50,000 to 100,000 cells. The conditions of the mRNA transfection are summarized in table S4.

### Flow cytometry

Twenty-four hours after the transfection, the cells were detached, filtered, and then analyzed with a BD FACSAria II (BD Biosciences). The fluorescence of hmAG1, hmKO2, tagBFP, and hdKRed was measured using a blue laser with a FITC filter, a green laser with a PE filter, a violet laser with a Pacific Blue filter, and a violet laser with a Qdot 605 filter, respectively. From the analyzed cell populations (10,000 counts), dead cells and debris were gated out according to forward scatter and side scatter, and noise in the hmKO2 measurement was removed on the basis of the height and area of the signal intensity. Using the fluorescence intensities of a cell, the expression from the synthetic mRNAs was evaluated with the indices defined in this study: fluorescence ratio, reporter expression, and relative expression (see Supplementary Text for details). In the experiments using a set of four reporter mRNAs, the fluorescence intensities were compensated to determine the levels of the four fluorescent proteins, as described previously (*38*). Briefly, a spillover matrix of four fluorescent proteins into the four detection channels was experimentally determined for each transfection series on each cell type. The values in the matrix were obtained by fitting flow cytometric data from the cells transfected with either one of the four reporter mRNAs (control mRNAs) or untransfected cells with gnuplot (www.gnuplot.info). The fluorescence intensities in single cells were compensated with the compensation matrix, which is the inverse of the spillover matrix, and then used to calculate the fluorescence ratios.

### Data fitting

Data fitting shown in Fig. 3C was performed with gnuplot based on a nonlinear least-squares method. In the evaluation of ρ*^i^* (*i* = 1 to 5), the relative expressions of a series of five-slot mRNAs, one to three slots of which responded to the identical miRNA (Fig. 3A), were experimentally determined. Then, 25 or 26 data points of the series were subjected to data fitting by eq. S4 (Supplementary Text) with the five parameters (ρ*^i^*; *i* = 1 to 5) for each of the four model miRNAs. The mean values of the resulting parameters (20 data points in total) from three independent analyses were used for further data fitting to examine the relationship between the distance of a slot from the start codon and the sensitivity of the slot to an miRNA. According to eq. S5 (Supplementary Text) and an exponential model, *k_i_* = *d_i_^x^*, a global variant, *x*, and four local variants: −log(ρ_miR-34a-5p_), −log(ρ_miR-17-5p_), −log(ρ_miR-92a-3p_), and −log(ρ_miR-21-5p_), were fitted to the mean values of the dataset.

### Normalization of miRNA activity profiles

Reporter expressions (eq. S2 in Supplementary Text) of 270 single-slot mRNAs in each cell type were determined from the compensated datasets of the flow cytometry. Notably, only one-third of miRNA species expressed in a cell repress the expression of their corresponding reporter mRNAs (*24*). This observation suggests that two-thirds of single-slot mRNAs used in this study were not affected by their corresponding miRNAs. Using reporter expressions in HeLa cells as a standard, the expressions of hmAG1, tagBFP, and hdKRed in each cell type were independently corrected according to simple linear regression without the intercept term to obtain bias-corrected expressions. Then, the mean and SD of the bias caused by differences in the reporter fluorescent proteins was heuristically set to 0.5 and 0.15, respectively.

### Statistical analysis

Data presented with error bars are the means ± SD from three independent experiments (*n* = 3). Pearson product-moment correlation coefficients (*r*), determination coefficients (*R*^2^), and root mean square errors between the model and experimental observations were calculated using the means of three observations (*n* = 3). PCA was performed using the activity profiles of 270 miRNAs in eight cell types (Fig. 4D) and those of 54 miRNAs in hiPSCs in nine conditions (Fig. 5D).

## Supplementary Material

http://advances.sciencemag.org/cgi/content/full/5/8/eaax0835/DC1

Download PDF

## SUPPLEMENTARY MATERIALS

Supplementary material for this article is available at http://advances.sciencemag.org/cgi/content/full/5/8/eaax0835/DC1 Supplementary Text Fig. S1. The effect of miRNA inhibitors on the measurement. Fig. S2. The positional effect of miRNA target sequences in synthetic mRNAs for miRNA-mediated repression. Fig. S3. Steps in the normalization of the screening data in Fig. 4. Fig. S4. Live cell classification with four synthetic mRNAs. Fig. S5. Tracking of hiPSCs with four five-slot mRNAs. Table S1. 5′UTR sequences of the five-slot mRNAs used in this study. Table S2. 5′UTR sequences of the single-slot mRNAs used in this study. Table S3. List of primers, single-stranded oligo DNAs, and plasmids for PCR amplification. Table S4. List of experimental conditions.
