# Supplementary material for: Numerical operations in living cells by programmable RNA devices
Source: Sci Adv. 2019 Aug 21;5(8):eaax0835. doi: 10.1126/sciadv.aax0835 (PMC6703868; doi:10.1126/sciadv.aax0835)
Supplement: http://advances.sciencemag.org/cgi/content/full/5/8/eaax0835/DC1 [file supp_5_8_eaax0835__index.html]

Science Advances | Science AdvancesAAASSearchScience AdvancesMenu

## Supplementary Materials

**This PDF file includes:**

- Supplementary Text
- Fig. S1. The effect of miRNA inhibitors on the measurement.
- Fig. S2. The positional effect of miRNA target sequences in synthetic mRNAs for miRNA-mediated repression.
- Fig. S3. Steps in the normalization of the screening data in Fig. 4.
- Fig. S4. Live cell classification with four synthetic mRNAs.
- Fig. S5. Tracking of hiPSCs with four five-slot mRNAs.
- Table S1. 5′UTR sequences of the five-slot mRNAs used in this study.
- Table S2. 5′UTR sequences of the single-slot mRNAs used in this study.
- Table S3. List of primers, single-stranded oligo DNAs, and plasmids for PCR amplification.
- Table S4. List of experimental conditions.

Download PDF

**Files in this Data Supplement:**

- Adobe PDF - aax0835\_SM.pdf
