# Supplementary material for: Numerical operations in living cells by programmable RNA devices
Source: Sci Adv. 2019 Aug 21;5(8):eaax0835. doi: 10.1126/sciadv.aax0835 (PMC6703868; doi:10.1126/sciadv.aax0835)
Supplement: Download PDF [file aax0835_SM.pdf]

Science Advances
AAAS

advances.sciencemag.org/cgi/content/full/5/8/eaax0835/DC1

Supplementary Materials for

Numerical operations in living cells by programmable RNA devices

Kei Endo*, Karin Hayashi, Hirohide Saito*

*Corresponding author. Email: kei-endo@k.u-tokyo.ac.jp (K.E.); hirohide.saito@cira.kyoto-u.ac.jp (H.S.)

Published 21 August 2019, *Sci. Adv.* **5**, eaax0835 (2019)
DOI: 10.1126/sciadv.aax0835

This PDF file includes:

Supplementary Text
Fig. S1. The effect of miRNA inhibitors on the measurement.
Fig. S2. The positional effect of miRNA target sequences in synthetic mRNAs for miRNA-mediated repression.
Fig. S3. Steps in the normalization of the screening data in Fig. 4.
Fig. S4. Live cell classification with four synthetic mRNAs.
Fig. S5. Tracking of hiPSCs with four five-slot mRNAs.
Table S1. 5′UTR sequences of the five-slot mRNAs used in this study.
Table S2. 5′UTR sequences of the single-slot mRNAs used in this study.
Table S3. List of primers, single-stranded oligo DNAs, and plasmids for PCR amplification.
Table S4. List of experimental conditions.

Supplementary Text

Indices for reporter fluorescent protein expressions from synthetic mRNAs

The fluorescence intensity of a cell transfected with fluorescent protein-encoding mRNAs typically ranged over 100 fold, and the fluorescent intensities of single cells co-transfected with two mRNAs coding distinct fluorescent proteins showed high consistency (*20*). Thus, throughout the analyses, we calculated ***fluorescence ratio***, which is the ratio of the intensities of two fluorescent proteins in a cell. Here is an example

$$Fluorescence\ ratio = \frac{[hmAG1]}{[tagBFP]} \tag{1}$$

[hmAG1] and [tagBFP] describe the fluorescence intensity of hmAG1 and tagBFP protein, respectively, in each analyzed cell. To compare the behavior of the designed mRNAs, we define ***reporter expression*** as the normalized geometric mean of ***fluorescence ratio*** in a transfected population. That is, the fluorescence intensity from a reporter mRNA is divided by the fluorescence intensity from the transfection control mRNAs encoding a different fluorescent protein. Then, the mean ratio of the fluorescence from the reporter mRNA is normalized by the fluorescence intensity of the control mRNA that encodes the same fluorescent protein as the reporter and is free from miRNA target sequences. In the proof-of-principle experiments shown in Figure 2 and 3, a five-slot reporter mRNA encodes hmAG1 and the transfection control mRNA encodes tagBFP. Thus, ***reporter expression*** in these analyses is as follows

$$Reporter\ expression = \left\langle \frac{[hmAG1]_{reporter}}{[tagBFP]} \right\rangle \bigg/ \left\langle \frac{[hmAG1]_{control}}{[tagBFP]} \right\rangle \tag{2}$$

In the screening experiments shown in Figures 4 and 5, single-slot reporter mRNAs encode hmAG1, tagBFP or hdKRed, and the transfection control mRNA encodes hmKO2.

We observed that mRNAs composed of different 5' UTR sequences expressed different levels of reporter proteins regardless of miRNA activity. To evaluate the effect of miRNAs on the designed mRNAs accurately, we define ***relative expression*** as ***reporter expression*** normalized by ***reporter expression*** in the presence of all inhibitors for miRNAs to which the reporter mRNA responds. That is, ***relative expression*** of the designed mRNA in the presence of all corresponding inhibitors is set to 1

$$Relative\ expression = \frac{Reporter\ expression_{condition\_of\_interest}}{Reporter\ expression_{w/\_corresponding\_inhibitors}} \quad (3)$$

A model for a multivariate linear combination

We estimated ***relative expression*** of the designed multi-slot mRNAs. The additivity of the repression by multiple miRNAs is supported by the results in Figure 2C. Thus, we define ***estimated expression*** as the product of ***relative expression*** affected by each miRNA at each slot. If we represent ***relative expression*** by a miRNA at slot-***i*** as $\boldsymbol{\rho^i}$, then ***estimated expression*** is described as shown in Figure 3A

$$Estimated\ expression = \prod_i \rho^i \quad (4)$$

With Eq. 4 and a dataset of ***relative expression*** from a five-slot mRNA series responding to one of the four model miRNAs (*e.g.* Fig. 3A; see Supplementary Table S1 for the 5' UTR sequences), we performed data fitting by the least squares method to determine $\boldsymbol{\rho^i}$ ($\boldsymbol{i}$ = 1–5) as shown in fig. S2B.

The activity of an miRNA in a cell should be represented as a unique value. In this study, the activity of miRNA-***a*** is defined as the inverse of ***relative expression*** of an ideal mRNA responding to miRNA-***a*** and denoted as $-\log(\boldsymbol{\rho_a})$. Based on the activity, the level of repression on a designed reporter mRNA is tuned by the position of the miRNA target sequence in the mRNA.

Thus, the repression by miRNA-$\boldsymbol{a}$ at slot-$\boldsymbol{i}$ is described as ***local repression***, $-\log(\boldsymbol{\rho}^{\boldsymbol{i}}_{\boldsymbol{a}})$, which depends on ideal repression, $-\log(\boldsymbol{\rho}_{\boldsymbol{a}})$, and the position of the slot, $\boldsymbol{i}$. Using a position-dependent tuning factor, $\boldsymbol{k}_{\boldsymbol{i}}$ (Fig. 1B), the positional effect at slot-$\boldsymbol{i}$ is hypothesized as follows

$$Local\ repression: -\log(\rho_a^i) = -k_i \cdot \log(\rho_a) \tag{5}$$

According to the results shown in fig. S2, the tuning factor is a function of the distance of the slot from the start codon, $\boldsymbol{d}_{\boldsymbol{i}}$. Simultaneous data fitting by the least squares method shown in Figure 3C leads us to a model and a global constant that explain the positional effect of the slot independently of the miRNA species

$$k_i = d_i^{-0.56} \tag{6}$$

Eq. 6 indicates that the target sequence to miRNA-$\boldsymbol{a}$ of the ideal mRNA is located at one nucleotide from the start codon ($\boldsymbol{d}$ = 1). According to Eq. 4, 5 and 6, the expression from a synthetic reporter mRNA responding to multiple miRNAs is estimated by a linear combination of the multiple miRNA activities in a cell

$$\log(estimated\ expression) = \sum_i \log(\rho_i^i) = \sum_i k_i \cdot \log(\rho_i) = \sum_i d_i^{-0.56} \cdot \log(\rho_i) \tag{7}$$

Here, $\boldsymbol{\rho}_{\boldsymbol{i}}$ denotes the activity of an miRNA, the target sequence of which is located at slot-$\boldsymbol{i}$. In Eq. 7, coefficients for multiple miRNA activities (tuning factors) are always positive. Then, the ratio of ***estimated expression*** extends the coefficient range to negative values

$$\begin{aligned} &\log(estimated\ \ fluorescence\ ratio) \\ &\quad = \log(estimated\ expression_{mRNA[i]}) - \log(estimated\ expression_{mRNA[j]}) \\ &\quad = \sum_i d_i^{-0.56} \cdot \log(\rho_i) + \sum_j (-d_j^{-0.56}) \cdot \log(\rho_j) \end{aligned} \tag{8}$$

Here, mRNA[***i***] and mRNA[***j***] indicate two distinct multi-slot mRNAs that encode different fluorescent proteins. Furthermore, the use of the ratio from two reporter mRNAs enables us to omit the transfectional control mRNA. Thus, the transfection of two synthetic reporter mRNAs results in one synthetic parameter that recapitulates a miRNA activity profile in a living cell according to the linear combinations described in Eq. 8.

Design of five-slot mRNA sets

First, the normalized miRNA activity profiles (270 miRNAs in eight cell types) were subjected to multivariate statistical analysis to select miRNAs that were expected to have high potential to classify the eight different cell types shown in Figure 4D. According to the results of the principal component analysis (PCA) and cluster analysis with Ward's method, the miRNAs, the coefficients (contribution) of which for the first two components calculated by the PCA were the highest in each of the 49 clusters made by the cluster analysis, were selected as candidates. The profiles of the 49 miRNAs were further analyzed by PCA. Then, 26 of the 49 miRNAs were selected according to the absolute values of the contribution to the first two components calculated in the second PCA.

Next, the classification of the eight cell types was simulated based on the estimated expressions of a five-slot mRNA. The estimated expressions in the eight cell types of a systematically designed five-slot mRNA that responded to some of the selected 26 miRNAs were calculated. The mRNAs with an estimated expression in a cell type lower than 0.05 were screened out to avoid noise by the cellular autofluorescence. The design of the five slots that showed the highest variance among the cell types was adopted as the 5' UTR of the first reporter mRNA (encoding hmAG1, for example). To screen the 5' UTR of the second mRNA (hmKO2), the ratio of the estimated expressions from the systematically designed five-slot mRNAs to that from the determined first mRNA was systematically calculated. The design of the five slots that showed the highest variance of the ratio among the cell types was adopted. To screen the 5' UTR of the third mRNA (tagBFP), the correlation coefficient (R) and the two-dimensional variance (V) of the ratio of the estimated expressions from the first and the second mRNAs with the estimated expression from the systematically designed five-slot mRNAs was systematically calculated. The design of the 5' UTR was determined based on the criteria $r < 0.3$ and $\max(V)$. Finally, using the

estimated expressions from three determined mRNAs and the systematically designed five-slot mRNAs as a fourth value, live cell classification with a set of four mRNAs was simulated (Fig. 4F). The mRNA set variance of two fluorescence ratios among the eight cell types was adopted.

Another set for tracking changes in hiPSCs was designed in a similar way except 11 out of 54 miRNAs were selected and used for the simulation and calculation (Fig. 5).

Supplementary Figures

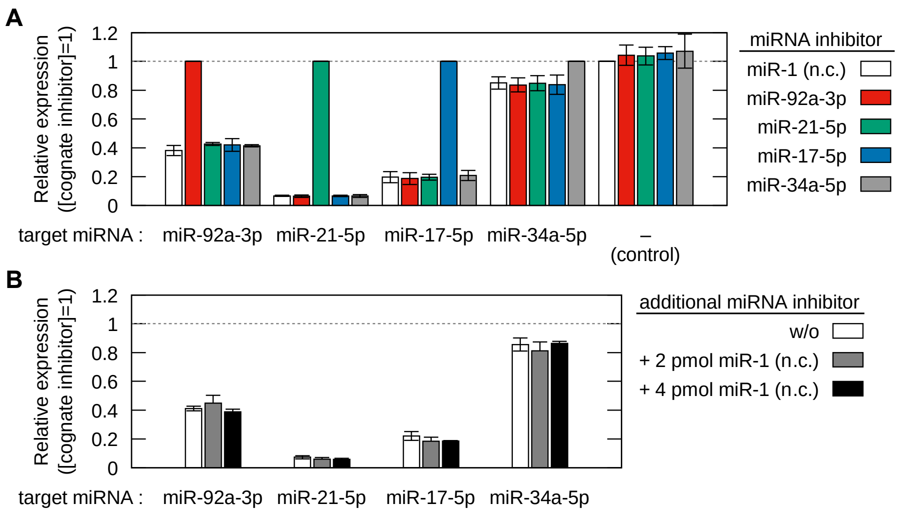

Fig. S1. The effect of miRNA inhibitors on the measurement. (**A**) Off-target effects of the miRNA inhibitors used in this study. The relative expression of single-slot mRNAs responding to the indicated miRNA (target miRNA) was measured in the presence of inhibitors for one of the four model miRNAs in this study or miR-1. The miR-1 inhibitor was used as a negative control. The relative expressions in the presence of the cognate inhibitor was set to 1. The 5' UTR of the control mRNA had no miRNA target sequence. Error bars indicate the mean ± standard deviation (n=3). In this condition, miRNA inhibitors showed no significant off-target effects. The relative expressions were measured in the presence of 2 pmol miRNA inhibitor. (**B**) Effect of the total amount of miRNA inhibitors. White bars (w/o) show the relative expressions of single-slot mRNAs measured in the same condition as in **A,** where the expression with the corresponding miRNA inhibitor was set to 1. Gray and black bars show the relative expressions measured in the presence of an additional 2 and 4 pmol miR-1 inhibitor, respectively. In total, 4 and 6 pmol miRNA inhibitors with reporter mRNAs were transfected into HeLa cells. Error bars indicate the mean ± SD (*N*=3). In these conditions, the total amount of miRNA inhibitors hardly affected the relative expression.

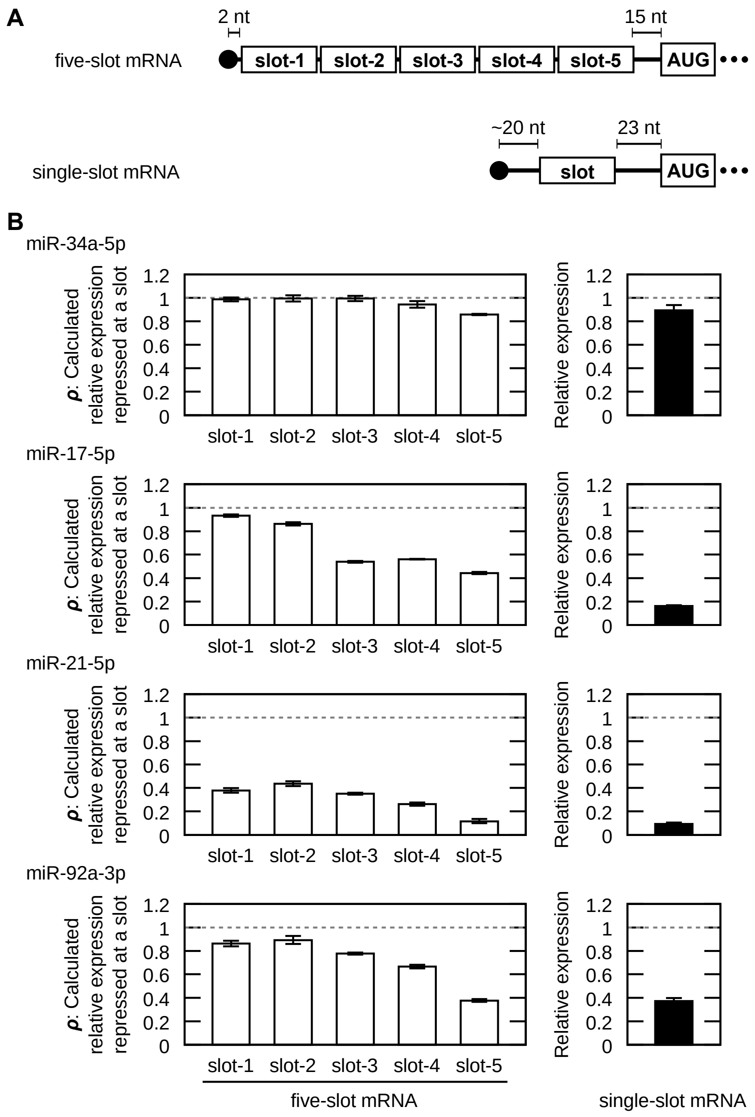

Fig. S2. The positional effect of miRNA target sequences in synthetic mRNAs for miRNA-mediated repression. (**A**) The 5' UTR designs of a five-slot (top) and a single-slot (bottom) mRNA. Boxes labeled with slots are the regions for the miRNA target sequences. Black circles and AUG depict a cap structure and the start codon of synthetic mRNAs, respectively. nt, nucleotides. (**B**) Repression by the four model miRNAs at different slots. The relative expressions of the five-slot mRNAs repressed by the indicated miRNA at the indicated slot (ρ values, gray bars) were obtained by fitting the results from the series of five-slot mRNAs (Fig. 3A, see Supplementary Table S1 for the sequences). The relative expression of single-slot mRNAs (black bars) was determined experimentally. Error bars indicate the mean ± SD (*N*=3). An outlier was excluded in the single-slot mRNA for miR-21.

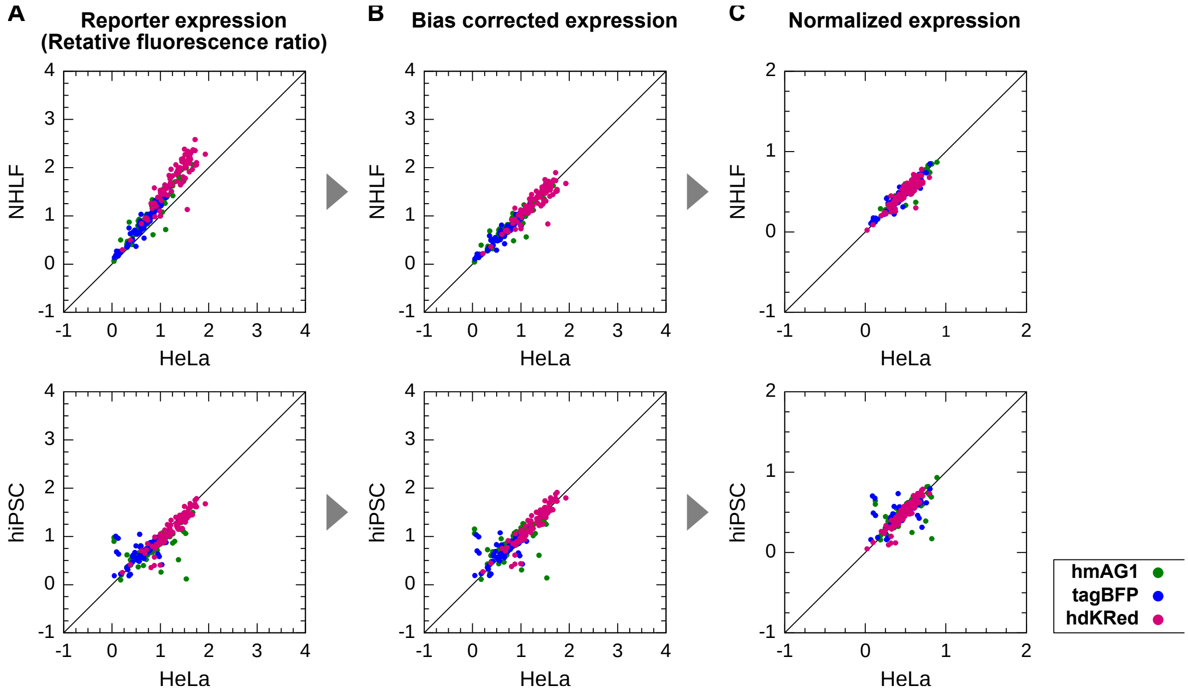

Fig. S3. Steps in the normalization of the screening data in Fig. 4. Three panels on the top show the normalization process for NHLFs and those on the bottom for hiPSCs based on expression data obtained from HeLa cells. Dots in a plot indicate the reporter expressions (**A**), the bias corrected expression (**B**), and the normalized expression (**C**) of 270 single-slot mRNAs encoding hmAG1 (green), tagBFP (blue) and hdKRed (purple). See Supplementary Text for details.

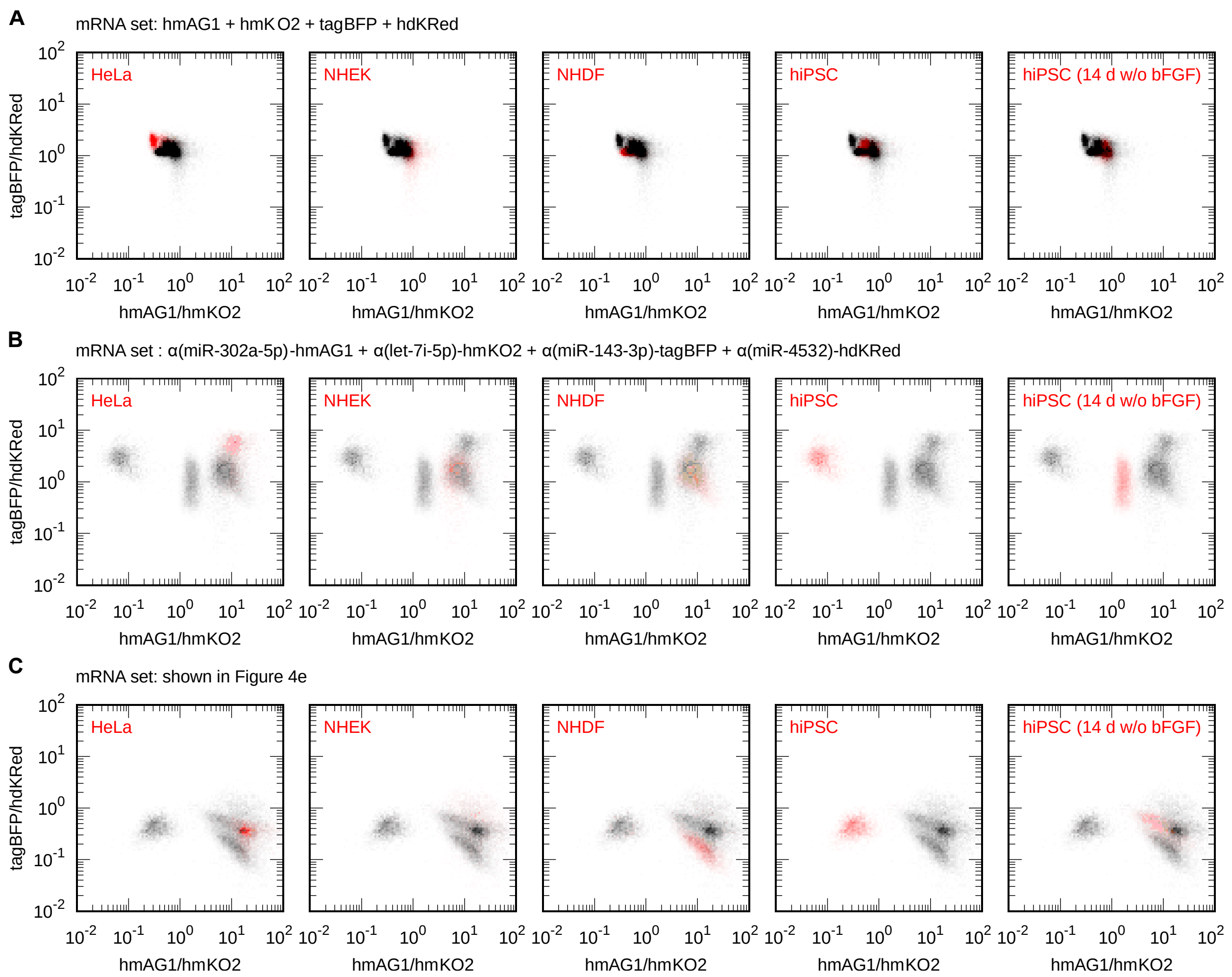

Fig. S4. Live cell classification with four synthetic mRNAs. Live cell classification with the transfection of four reporter mRNAs and flow cytometry. The indicated cells were transfected with the set of control mRNAs (**A**), set of four single-slot mRNAs (**B**), and set of four five-slot mRNAs used for tracking changes in hiPSCs (**C**, shown in Fig. 5E). In the principal component analysis shown in Fig. 4D, miR-302a-5p and let-7i-5p are the most weighted in component 1, and miR-143-3p and miR-4532 in component 2. The density of the cells analyzed by flow cytometry was plotted on two fluorescence ratios: hmAG1/hmKO2 and tagBFP/hdKRed. The indicated cells presented in red density and other cells in black density were merged.

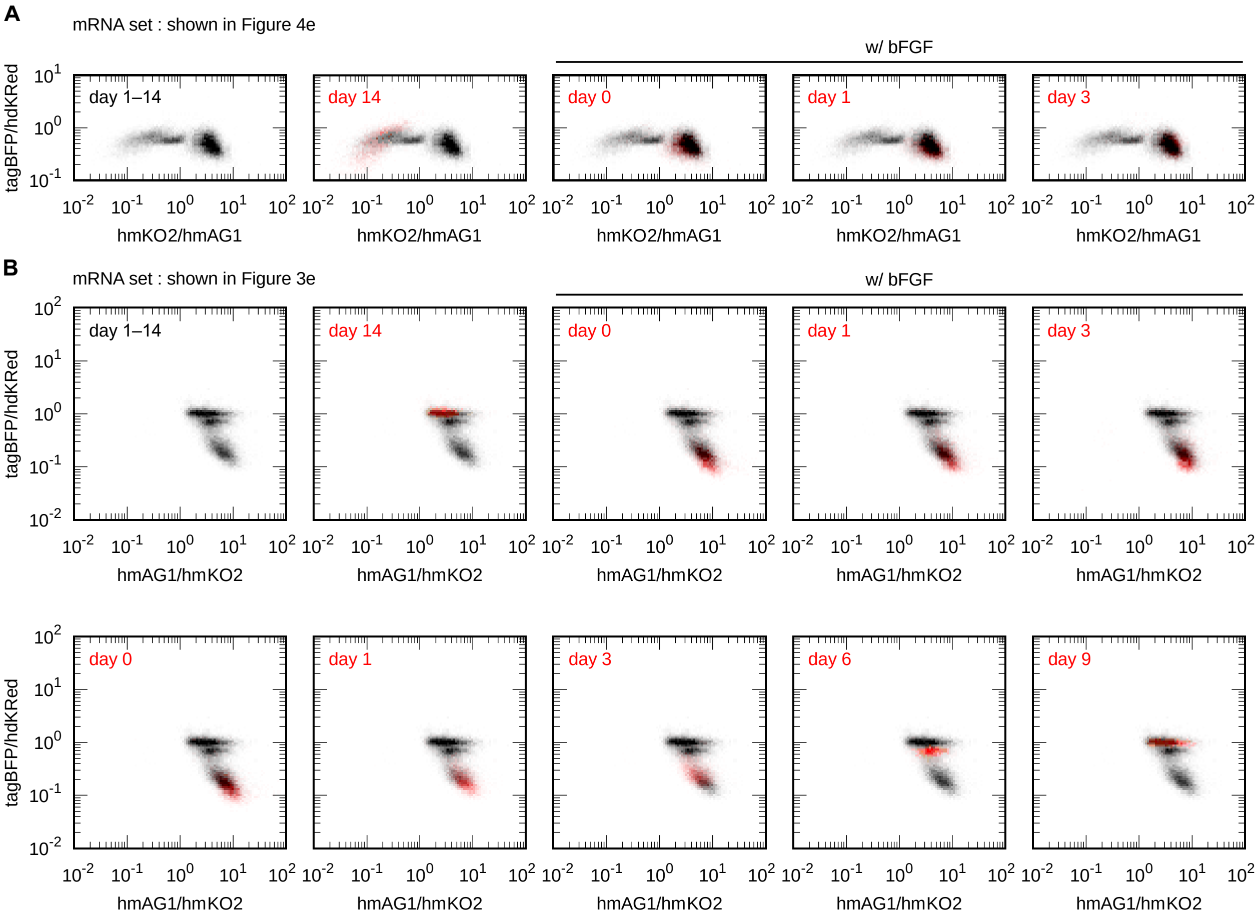

Fig. S5. Tracking of hiPSCs with four five-slot mRNAs. (**A**) Density plots of transfected hiPSCs cultured without bFGF for 14 days and those with bFGF for 0, 1, and 3 days before the transfection of the mRNA set for tracking hiPSCs changes (shown in Figure 5E). All density plots including those in Figure 5G were merged (day 1-14). The density of the indicated conditions is presented in red density over the merged plots. (**B**) Tracking of hiPSCs with the mRNAs set used in the classification of cell types (shown in Fig. 4E) with bFGF (top, 0, 1, and 3 days) or without bFGF. The X-axis of the plots represents hmAG1/hmKO2, as shown in Figure 4G and fig. S4. The transfected iPSCs at the indicated time points are presented in red density over the merged plots.

Supplementary Tables

Table S1. 5′UTR sequences of the five-slot mRNAs used in this study.

Figure	slot-1	slot-2	slot-3	slot-4	slot-5	5' UTR sequence*[1]	Protein
control	-	-	-	-	-	GGCCGCUUGAAGUCUUUAAUUAAACCCGCUUGAAGUCUUUAAUUAAACGAACGGGCACGCUGACAAUUCAAGCACUCUGAUUUGACAAUUACAAGCACUCUGAUUUGACAAUUACACCGGUCGCCACCAUG	hmAG1
Fig. 2A	-	miR-17-5p	-	miR-92a-3p	-	GGCCGCUUGAAGUCUUUAAUUAAACUACCUGCACUGUAAGCACUUUGCGAACGGGCACGCUGACAAUUACAGGCCGGGACAAGUGCAAUAAAGCACUCUGAUUUGACAAUUACACCGGUCGCCACCAUG	hmAG1
Fig. 2A	miR-21-5p	-	-	-	miR-92a-3p	GGUCAACAUCAGUCUGAUAAGCUACCGCUUGAAGUCUUUAAUUAAACGAACGGGCACGCUGACAAUUAAGCACUCUGAUUUGACAAUUAACAGGCCGGGACAAGUGCAAUACACCGGUCGCCACCAUG	hmAG1
Fig. 2A	-	-	-	miR-21-5p	miR-17-5p	GGCCGCUUGAAGUCUUUAAUUAAACCCGCUUGAAGUCUUUAAUUAAACGAACGGGCACGCUGACAAUUUCAACAUCAGUCUGAUAAGCUACUACCUGCACUGUAAGCACUUUGCACCGGUCGCCACCAUG	hmAG1
Fig. 2A	-	miR-21-5p	-	miR-17-5p	-	GGCCGCUUGAAGUCUUUAAUUAAAUCAACAUCAGUCUGAUAAGCUACGAACGGGCACGCUGACAAUUCCUACCUGCACUGUAAGCACUUUGAAGCACUCUGAUUUGACAAUUACACCGGUCGCCACCAUG	hmAG1
Fig. 2A	-	miR-92a-3p	-	-	miR-34a-5p	GGCCGCUUGAAGUCUUUAAUUAAAACAGGCCGGGACAAGUGCAAUACGAACGGGCACGCUGACAAUUAAGCACUCUGAUUUGACAAUUAACAACCAGCUAAGACACUGCCACACCGGUCGCCACCAUG	hmAG1
Fig. 2A	miR-34a-5p	-	miR-34a-5p	miR-34a-5p	miR-92a-3p	GGACAACCAGCUAAGACACUGCCACCGCUUGAAGUCUUUAAUUAAAACAACCAGCUAAGACACUGCCAGACAACCAGCUAAGACACUGCCAACAGGCCGGGACAAGUGCAAUACACCGGUCGCCACCAUG	hmAG1
Fig. 2A	-	-	miR-17-5p	-	miR-34a-5p	GGCCGCUUGAAGUCUUUAAUUAAACCGCUUGAAGUCUUUAAUUAAACUACCUGCACUGUAAGCACUUUGGAAGCACUCUGAUUUGACAAUUAACAACCAGCUAAGACACUGCCACACCGGUCGCCACCAUG	hmAG1
Fig. 2A	-	miR-17-5p	miR-34a-5p	-	miR-34a-5p	GGCCGCUUGAAGUCUUUAAUUAAACUACCUGCACUGUAAGCACUUUGACAACCAGCUAAGACACUGCCAGAAGCACUCUGAUUUGACAAUUAACAACCAGCUAAGACACUGCCACACCGGUCGCCACCAUG	hmAG1
Fig. 2A	miR-34a-5p	-	miR-92a-3p	-	miR-17-5p	GGACAACCAGCUAAGACACUGCCACCGCUUGAAGUCUUUAAUUAAAGACAGGCCGGGACAAGUGCAAUAAAGCACUCUGAUUUGACAAUUACUACCUGCACUGUAAGCACUUUGCACCGGUCGCCACCAUG	hmAG1

Fig. 2A	miR-34a-5p	miR-92a-3p	-	miR-34a-5p	miR-17-5p	GGACAACCAGCUAAGACACUGCCAACAGGCCGGGACAAGUGCAAUACGAACGGGCACGCUGACAAUUACAACCAGCUAAGACACUGCCACUACCUGCACUGUAAGCACUUUGCACCGGUCGCCACCAUG	hmAG1
Fig. 2A	miR-17-5p	miR-21-5p	-	-	miR-34a-5p	GGCUACCUGCACUGUAAGCACUUUGUCAACAUCAGUCUGAUAAGCUACGAACGGGCACGCUGACAAUUAAGCACUCUGAUUUGACAAUUAACAACCAGCUAAGACACUGCCACACCGGUCGCCACCAUG	hmAG1
Fig. 2A	miR-21-5p	-	miR-92a-3p	miR-17-5p	-	GGUCAACAUCAGUCUGAUAAGCUACCGCUUGAAGUCUUUAAUUAAAGACAGGCCGGGACAAGUGCAAUACUACCUGCACUGUAAGCACUUUGAAGCACUCUGAUUUGACAAUUACACCGGUCGCCACCAUG	hmAG1
Fig. 3A	-	-	-	-	miR-34a-5p	GGCCGCUUGAAGUCUUUAAUUAAACCCGCUUGAAGUCUUUAAUUAAACGAACGGGCACGCUGACAAUUAAGCACUCUGAUUUGACAAUUAACAACCAGCUAAGACACUGCCACACCGGUCGCCACCAUG	hmAG1
Fig. 3A	-	-	-	miR-34a-5p	-	GGCCGCUUGAAGUCUUUAAUUAAACCCGCUUGAAGUCUUUAAUUAAACGAACGGGCACGCUGACAAUUACAACCAGCUAAGACACUGCCAAAGCACUCUGAUUUGACAAUUACACCGGUCGCCACCAUG	hmAG1
Fig. 3A	-	-	miR-34a-5p	-	-	GGCCGCUUGAAGUCUUUAAUUAAACCGCUUGAAGUCUUUAAUUAAAACAACCAGCUAAGACACUGCCAGAAGCACUCUGAUUUGACAAUUAAAGCACUCUGAUUUGACAAUUACACCGGUCGCCACCAUG	hmAG1
Fig. 3A	-	miR-34a-5p	-	-	-	GGCCGCUUGAAGUCUUUAAUUAAAACAACCAGCUAAGACACUGCCACGAACGGGCACGCUGACAAUUCAAGCACUCUGAUUUGACAAUUACAAGCACUCUGAUUUGACAAUUACACCGGUCGCCACCAUG	hmAG1
Fig. 3A	miR-34a-5p	-	-	-	-	GGACAACCAGCUAAGACACUGCCACCGCUUGAAGUCUUUAAUUAAACGAACGGGCACGCUGACAAUUCAAGCACUCUGAUUUGACAAUUACAAGCACUCUGAUUUGACAAUUACACCGGUCGCCACCAUG	hmAG1
Fig. 3A	-	-	-	miR-34a-5p	miR-34a-5p	GGCCGCUUGAAGUCUUUAAUUAAACCCGCUUGAAGUCUUUAAUUAAACGAACGGGCACGCUGACAAUUACAACCAGCUAAGACACUGCCAACAACCAGCUAAGACACUGCCACACCGGUCGCCACCAUG	hmAG1
Fig. 3A	-	-	miR-34a-5p	-	miR-34a-5p	GGCCGCUUGAAGUCUUUAAUUAAACCGCUUGAAGUCUUUAAUUAAAACAACCAGCUAAGACACUGCCAGAAGCACUCUGAUUUGACAAUUAACAACCAGCUAAGACACUGCCACACCGGUCGCCACCAUG	hmAG1
Fig. 3A	-	miR-34a-5p	-	-	miR-34a-5p	GGCCGCUUGAAGUCUUUAAUUAAAACAACCAGCUAAGACACUGCCACGAACGGGCACGCUGACAAUUAAGCACUCUGAUUUGACAAUUAACAACCAGCUAAGACACUGCCACACCGGUCGCCACCAUG	hmAG1
Fig. 3A	miR-34a-5p	-	-	-	miR-34a-5p	GGACAACCAGCUAAGACACUGCCACCGCUUGAAGUCUUUAAUUAAACGAACGGGCACGCUGACAAUUAAGCACUCUGAUUUGACAAUUAACAACCAGCUAAGACACUGCCACACCGGUCGCCACCAUG	hmAG1
Fig. 3A	-	-	miR-34a-5p	miR-34a-5p	-	GGCCGCUUGAAGUCUUUAAUUAAACCGCUUGAAGUCUUUAAUUAAAACAACCAGCUAAGACACUGCCAGACAACCAGCUAAGACACUGCCAAAGCACUCUGAUUUGACAAUUACACCGGUCGCCACCAUG	hmAG1

Fig. 3A	-	miR-34a-5p	-	miR-34a-5p	-	GGCCGCUUGAAGUCUUUAAUUAAAACAACCAGCUAAGACACUGCCACGAACGGGCACGCUGACAAUUACAACCAGCUAAGACACUGCCAAAGCACUCUGAUUUGACAAUUACACCGGUCGCCACCAUG	hmAG1
Fig. 3A	miR-34a-5p	-	-	miR-34a-5p	-	GGACAACCAGCUAAGACACUGCCACCGCUUGAAGUCUUUAAUUAAACGAACGGGCACGCUGACAAUUACAACCAGCUAAGACACUGCCAAAGCACUCUGAUUUGACAAUUACACCGGUCGCCACCAUG	hmAG1
Fig. 3A	-	miR-34a-5p	miR-34a-5p	-	-	GGCCGCUUGAAGUCUUUAAUUAAAACAACCAGCUAAGACACUGCCAACAACCAGCUAAGACACUGCCAGAAGCACUCUGAUUUGACAAUUAAAGCACUCUGAUUUGACAAUUACACCGGUCGCCACCAUG	hmAG1
Fig. 3A	miR-34a-5p	-	miR-34a-5p	-	-	GGACAACCAGCUAAGACACUGCCACCGCUUGAAGUCUUUAAUUAAAACAACCAGCUAAGACACUGCCAGAAGCACUCUGAUUUGACAAUUAAAGCACUCUGAUUUGACAAUUACACCGGUCGCCACCAUG	hmAG1
Fig. 3A	miR-34a-5p	miR-34a-5p	-	-	-	GGACAACCAGCUAAGACACUGCCAACAACCAGCUAAGACACUGCCACGAACGGGCACGCUGACAAUUCAAGCACUCUGAUUUGACAAUUACAAGCACUCUGAUUUGACAAUUACACCGGUCGCCACCAUG	hmAG1
Fig. 3A	-	miR-34a-5p	-	miR-34a-5p	miR-34a-5p	GGCCGCUUGAAGUCUUUAAUUAAAACAACCAGCUAAGACACUGCCACGAACGGGCACGCUGACAAUUACAACCAGCUAAGACACUGCCAACAACCAGCUAAGACACUGCCACACCGGUCGCCACCAUG	hmAG1
Fig. 3A	miR-34a-5p	-	-	miR-34a-5p	miR-34a-5p	GGACAACCAGCUAAGACACUGCCACCGCUUGAAGUCUUUAAUUAAACGAACGGGCACGCUGACAAUUACAACCAGCUAAGACACUGCCAACAACCAGCUAAGACACUGCCACACCGGUCGCCACCAUG	hmAG1
Fig. 3A	-	miR-34a-5p	miR-34a-5p	-	miR-34a-5p	GGCCGCUUGAAGUCUUUAAUUAAAACAACCAGCUAAGACACUGCCAACAACCAGCUAAGACACUGCCAGAAGCACUCUGAUUUGACAAUUAACAACCAGCUAAGACACUGCCACACCGGUCGCCACCAUG	hmAG1
Fig. 3A	miR-34a-5p	-	miR-34a-5p	-	miR-34a-5p	GGACAACCAGCUAAGACACUGCCACCGCUUGAAGUCUUUAAUUAAAACAACCAGCUAAGACACUGCCAGAAGCACUCUGAUUUGACAAUUAACAACCAGCUAAGACACUGCCACACCGGUCGCCACCAUG	hmAG1
Fig. 3A	miR-34a-5p	miR-34a-5p	-	-	miR-34a-5p	GGACAACCAGCUAAGACACUGCCAACAACCAGCUAAGACACUGCCACGAACGGGCACGCUGACAAUUAAGCACUCUGAUUUGACAAUUAACAACCAGCUAAGACACUGCCACACCGGUCGCCACCAUG	hmAG1
Fig. 3A	-	miR-34a-5p	miR-34a-5p	miR-34a-5p	-	GGCCGCUUGAAGUCUUUAAUUAAAACAACCAGCUAAGACACUGCCAACAACCAGCUAAGACACUGCCAGACAACCAGCUAAGACACUGCCAAAGCACUCUGAUUUGACAAUUACACCGGUCGCCACCAUG	hmAG1
Fig. 3A	miR-34a-5p	-	miR-34a-5p	miR-34a-5p	-	GGACAACCAGCUAAGACACUGCCACCGCUUGAAGUCUUUAAUUAAAACAACCAGCUAAGACACUGCCAGACAACCAGCUAAGACACUGCCAAAGCACUCUGAUUUGACAAUUACACCGGUCGCCACCAUG	hmAG1
Fig. 3A	miR-34a-5p	miR-34a-5p	-	miR-34a-5p	-	GGACAACCAGCUAAGACACUGCCAACAACCAGCUAAGACACUGCCACGAACGGGCACGCUGACAAUUACAACCAGCUAAGACACUGCCAAAGCACUCUGAUUUGACAAUUACACCGGUCGCCACCAUG	hmAG1

Fig. 3A	-	-	-	-	miR-17-5p	GGCCGCUUGAAGUCUUUAAUUAAACCCGCUUGAAGUCUUUAAUUAAACGAACGGGCACGCUGACAAUUCAAGCACUCUGAUUUGACAAUUACUACCUGCACUGUAAGCACUUUGCACCGGUCGCCACCAUG	hmAG1
Fig. 3A	-	-	-	miR-17-5p	-	GGCCGCUUGAAGUCUUUAAUUAAACCCGCUUGAAGUCUUUAAUUAAACGAACGGGCACGCUGACAAUUCCUACCUGCACUGUAAGCACUUUGAAGCACUCUGAUUUGACAAUUACACCGGUCGCCACCAUG	hmAG1
Fig. 3A	-	-	miR-17-5p	-	-	GGCCGCUUGAAGUCUUUAAUUAAACCCGCUUGAAGUCUUUAAUUAAACUACCUGCACUGUAAGCACUUUGAAGCACUCUGAUUUGACAAUUAAAGCACUCUGAUUUGACAAUUACACCGGUCGCCACCAUG	hmAG1
Fig. 3A	-	miR-17-5p	-	-	-	GGCCGCUUGAAGUCUUUAAUUAAACUACCUGCACUGUAAGCACUUUGCGAACGGGCACGCUGACAAUUCAAGCACUCUGAUUUGACAAUUACAAGCACUCUGAUUUGACAAUUACACCGGUCGCCACCAUG	hmAG1
Fig. 3A	miR-17-5p	-	-	-	-	GGCUACCUGCACUGUAAGCACUUUGCCGCUUGAAGUCUUUAAUUAAACGAACGGGCACGCUGACAAUUCAAGCACUCUGAUUUGACAAUUACAAGCACUCUGAUUUGACAAUUACACCGGUCGCCACCAUG	hmAG1
Fig. 3A	-	-	miR-17-5p	-	-	GGCCGCUUGAAGUCUUUAAUUAAACCGCUUGAAGUCUUUAAUUAAACUACCUGCACUGUAAGCACUUUGGAAGCACUCUGAUUUGACAAUUAAAGCACUCUGAUUUGACAAUUACACCGGUCGCCACCAUG	hmAG1
Fig. 3A	-	-	-	miR-17-5p	miR-17-5p	GGCCGCUUGAAGUCUUUAAUUAAACCCGCUUGAAGUCUUUAAUUAAACGAACGGGCACGCUGACAAUUCUACCUGCACUGUAAGCACUUUGCUACCUGCACUGUAAGCACUUUGCACCGGUCGCCACCAUG	hmAG1
Fig. 3A	-	-	miR-17-5p	-	miR-17-5p	GGCCGCUUGAAGUCUUUAAUUAAACCGCUUGAAGUCUUUAAUUAAACUACCUGCACUGUAAGCACUUUGGAAGCACUCUGAUUUGACAAUUACUACCUGCACUGUAAGCACUUUGCACCGGUCGCCACCAUG	hmAG1
Fig. 3A	-	miR-17-5p	-	-	miR-17-5p	GGCCGCUUGAAGUCUUUAAUUAAACUACCUGCACUGUAAGCACUUUGCGAACGGGCACGCUGACAAUUCAAGCACUCUGAUUUGACAAUUACUACCUGCACUGUAAGCACUUUGCACCGGUCGCCACCAUG	hmAG1
Fig. 3A	miR-17-5p	-	-	-	miR-17-5p	GGCUACCUGCACUGUAAGCACUUUGCCGCUUGAAGUCUUUAAUUAAACGAACGGGCACGCUGACAAUUCAAGCACUCUGAUUUGACAAUUACUACCUGCACUGUAAGCACUUUGCACCGGUCGCCACCAUG	hmAG1
Fig. 3A	-	-	miR-17-5p	miR-17-5p	-	GGCCGCUUGAAGUCUUUAAUUAAACCGCUUGAAGUCUUUAAUUAAACUACCUGCACUGUAAGCACUUUGGCUACCUGCACUGUAAGCACUUUGAAGCACUCUGAUUUGACAAUUACACCGGUCGCCACCAUG	hmAG1
Fig. 3A	-	miR-17-5p	-	miR-17-5p	-	GGCCGCUUGAAGUCUUUAAUUAAACUACCUGCACUGUAAGCACUUUGCGAACGGGCACGCUGACAAUUCCUACCUGCACUGUAAGCACUUUGAAGCACUCUGAUUUGACAAUUACACCGGUCGCCACCAUG	hmAG1
Fig. 3A	miR-17-5p	-	-	miR-17-5p	-	GGCUACCUGCACUGUAAGCACUUUGCCGCUUGAAGUCUUUAAUUAAACGAACGGGCACGCUGACAAUUCCUACCUGCACUGUAAGCACUUUGAAGCACUCUGAUUUGACAAUUACACCGGUCGCCACCAUG	hmAG1

Fig. 3A	-	miR-17-5p	miR-17-5p	-	-	GGCCGCUUGAAGUCUUUAAUUAAACUACCUGCACUGUAAGCACUUUGCUACCUGCACUGUAAGCACUUUGGAAGCACUCUGAUUUGACAAUUAAAGCACUCUGAUUUGACAAUUACACCGGUCGCCACCAUG	hmAG1
Fig. 3A	-	miR-17-5p	miR-17-5p	-	-	GGCCGCUUGAAGUCUUUAAUUAAACUACCUGCACUGUAAGCACUUUGCUACCUGCACUGUAAGCACUUUGGAAGCACUCUGAUUUGACAAUUAAAGCACUCUGAUUUGACAAUUACACCGGUCGCCACCAUG	hmAG1
Fig. 3A	miR-17-5p	miR-17-5p	-	-	-	GGCUACCUGCACUGUAAGCACUUUGCUACCUGCACUGUAAGCACUUUGCGAACGGGCACGCUGACAAUUCAAGCACUCUGAUUUGACAAUUACAAGCACUCUGAUUUGACAAUUACACCGGUCGCCACCAUG	hmAG1
Fig. 3A	-	miR-17-5p	-	miR-17-5p	miR-17-5p	GGCCGCUUGAAGUCUUUAAUUAAACUACCUGCACUGUAAGCACUUUGCGAACGGGCACGCUGACAAUUCUACCUGCACUGUAAGCACUUUGCUACCUGCACUGUAAGCACUUUGCACCGGUCGCCACCAUG	hmAG1
Fig. 3A	miR-17-5p	-	-	miR-17-5p	miR-17-5p	GGCUACCUGCACUGUAAGCACUUUGCCGCUUGAAGUCUUUAAUUAAACGAACGGGCACGCUGACAAUUCUACCUGCACUGUAAGCACUUUGCUACCUGCACUGUAAGCACUUUGCACCGGUCGCCACCAUG	hmAG1
Fig. 3A	-	miR-17-5p	miR-17-5p	-	miR-17-5p	GGCCGCUUGAAGUCUUUAAUUAAACUACCUGCACUGUAAGCACUUUGCUACCUGCACUGUAAGCACUUUGGAAGCACUCUGAUUUGACAAUUACUACCUGCACUGUAAGCACUUUGCACCGGUCGCCACCAUG	hmAG1
Fig. 3A	miR-17-5p	-	miR-17-5p	-	miR-17-5p	GGCUACCUGCACUGUAAGCACUUUGCCGCUUGAAGUCUUUAAUUAAACUACCUGCACUGUAAGCACUUUGGAAGCACUCUGAUUUGACAAUUACUACCUGCACUGUAAGCACUUUGCACCGGUCGCCACCAUG	hmAG1
Fig. 3A	miR-17-5p	miR-17-5p	-	-	miR-17-5p	GGCUACCUGCACUGUAAGCACUUUGCUACCUGCACUGUAAGCACUUUGCGAACGGGCACGCUGACAAUUCAAGCACUCUGAUUUGACAAUUACUACCUGCACUGUAAGCACUUUGCACCGGUCGCCACCAUG	hmAG1
Fig. 3A	miR-17-5p	-	miR-17-5p	miR-17-5p	-	GGCUACCUGCACUGUAAGCACUUUGCCGCUUGAAGUCUUUAAUUAAACUACCUGCACUGUAAGCACUUUGGCUACCUGCACUGUAAGCACUUUGAAGCACUCUGAUUUGACAAUUACACCGGUCGCCACCAUG	hmAG1
Fig. 3A	miR-17-5p	-	miR-17-5p	miR-17-5p	-	GGCUACCUGCACUGUAAGCACUUUGCCGCUUGAAGUCUUUAAUUAAACUACCUGCACUGUAAGCACUUUGGCUACCUGCACUGUAAGCACUUUGAAGCACUCUGAUUUGACAAUUACACCGGUCGCCACCAUG	hmAG1
Fig. 3A	miR-17-5p	miR-17-5p	-	miR-17-5p	-	GGCUACCUGCACUGUAAGCACUUUGCUACCUGCACUGUAAGCACUUUGCGAACGGGCACGCUGACAAUUCCUACCUGCACUGUAAGCACUUUGAAGCACUCUGAUUUGACAAUUACACCGGUCGCCACCAUG	hmAG1
Fig. 3A	-	-	-	-	miR-21-5p	GGCCGCUUGAAGUCUUUAAUUAAACCCGCUUGAAGUCUUUAAUUAAACGAACGGGCACGCUGACAAUUAAGCACUCUGAUUUGACAAUUAUCAACAUCAGUCUGAUAAGCUACACCGGUCGCCACCAUG	hmAG1
Fig. 3A	-	-	-	miR-21-5p	-	GGCCGCUUGAAGUCUUUAAUUAAACCCGCUUGAAGUCUUUAAUUAAACGAACGGGCACGCUGACAAUUUCAACAUCAGUCUGAUAAGCUAAAGCACUCUGAUUUGACAAUUACACCGGUCGCCACCAUG	hmAG1

Fig. 3A	-	-	miR-21-5p	-	-	GGCCGCUUGAAGUCUUUAAUUAAACCGCUUGAAGUCUUUAAUUAAA UCAACAUCAGUCUGAUAAGCUAGAAGCACUCUGAUUUGACAAUUAA AGCACUCUGAUUUGACAAUUACACCGGUCGCCACCAUG	hmAG1
Fig. 3A	-	miR-21-5p	-	-	-	GGCCGCUUGAAGUCUUUAAUUAAAUCAACAUCAGUCUGAUAAGCUA CGAACGGGCACGCUGACAAUUCAAGCACUCUGAUUUGACAAUUACA AGCACUCUGAUUUGACAAUUACACCGGUCGCCACCAUG	hmAG1
Fig. 3A	miR-21-5p	-	-	-	-	GGUCAACAUCAGUCUGAUAAGCUACCGCUUGAAGUCUUUAAUUAAA CGAACGGGCACGCUGACAAUUCAAGCACUCUGAUUUGACAAUUACA AGCACUCUGAUUUGACAAUUACACCGGUCGCCACCAUG	hmAG1
Fig. 3A	-	-	-	miR-21-5p	miR-21-5p	GGCCGCUUGAAGUCUUUAAUUAAACCCGCUUGAAGUCUUUAAUUAA ACGAACGGGCACGCUGACAAUUUCAACAUCAGUCUGAUAAGCUAUC AACAUCAGUCUGAUAAGCUACACCGGUCGCCACCAUG	hmAG1
Fig. 3A	-	-	miR-21-5p	-	miR-21-5p	GGCCGCUUGAAGUCUUUAAUUAAACCGCUUGAAGUCUUUAAUUAAA UCAACAUCAGUCUGAUAAGCUAGAAGCACUCUGAUUUGACAAUUAU CAACAUCAGUCUGAUAAGCUACACCGGUCGCCACCAUG	hmAG1
Fig. 3A	-	miR-21-5p	-	-	miR-21-5p	GGCCGCUUGAAGUCUUUAAUUAAAUCAACAUCAGUCUGAUAAGCUA CGAACGGGCACGCUGACAAUUAAGCACUCUGAUUUGACAAUUAUCA ACAUCAGUCUGAUAAGCUACACCGGUCGCCACCAUG	hmAG1
Fig. 3A	miR-21-5p	-	-	-	miR-21-5p	GGUCAACAUCAGUCUGAUAAGCUACCGCUUGAAGUCUUUAAUUAAA CGAACGGGCACGCUGACAAUUAAGCACUCUGAUUUGACAAUUAUCA ACAUCAGUCUGAUAAGCUACACCGGUCGCCACCAUG	hmAG1
Fig. 3A	-	-	miR-21-5p	miR-21-5p	-	GGCCGCUUGAAGUCUUUAAUUAAACCGCUUGAAGUCUUUAAUUAAA UCAACAUCAGUCUGAUAAGCUAGUCAACAUCAGUCUGAUAAGCUAA AGCACUCUGAUUUGACAAUUACACCGGUCGCCACCAUG	hmAG1
Fig. 3A	-	miR-21-5p	-	miR-21-5p	-	GGCCGCUUGAAGUCUUUAAUUAAAUCAACAUCAGUCUGAUAAGCUA CGAACGGGCACGCUGACAAUUUCAACAUCAGUCUGAUAAGCUAAAG CACUCUGAUUUGACAAUUACACCGGUCGCCACCAUG	hmAG1
Fig. 3A	miR-21-5p	-	-	miR-21-5p	-	GGUCAACAUCAGUCUGAUAAGCUACCGCUUGAAGUCUUUAAUUAAA CGAACGGGCACGCUGACAAUUUCAACAUCAGUCUGAUAAGCUAAAG CACUCUGAUUUGACAAUUACACCGGUCGCCACCAUG	hmAG1
Fig. 3A	-	miR-21-5p	miR-21-5p	-	-	GGCCGCUUGAAGUCUUUAAUUAAAUCAACAUCAGUCUGAUAAGCUA UCAACAUCAGUCUGAUAAGCUAGAAGCACUCUGAUUUGACAAUUAA AGCACUCUGAUUUGACAAUUACACCGGUCGCCACCAUG	hmAG1
Fig. 3A	miR-21-5p	-	miR-21-5p	-	-	GGUCAACAUCAGUCUGAUAAGCUACCGCUUGAAGUCUUUAAUUAAA UCAACAUCAGUCUGAUAAGCUAGAAGCACUCUGAUUUGACAAUUAA AGCACUCUGAUUUGACAAUUACACCGGUCGCCACCAUG	hmAG1
Fig. 3A	miR-21-5p	miR-21-5p	-	-	-	GGUCAACAUCAGUCUGAUAAGCUAUCAACAUCAGUCUGAUAAGCUA CGAACGGGCACGCUGACAAUUCAAGCACUCUGAUUUGACAAUUACA AGCACUCUGAUUUGACAAUUACACCGGUCGCCACCAUG	hmAG1

Fig. 3A	-	miR-21-5p	-	miR-21-5p	miR-21-5p	GGCCGCUUGAAGUCUUUAAUUAAAUCAACAUCAGUCUGAUAAGCUA<br>CGAACGGGCACGCUGACAAUUUCAACAUCAGUCUGAUAAGCUAUCA<br>ACAUCAGUCUGAUAAGCUACACCGGUCGCCACCAUG	hmAG1
Fig. 3A	miR-21-5p	-	-	miR-21-5p	miR-21-5p	GGUCAACAUCAGUCUGAUAAGCUACCGCUUGAAGUCUUUAAUUAAA<br>CGAACGGGCACGCUGACAAUUUCAACAUCAGUCUGAUAAGCUAUCA<br>ACAUCAGUCUGAUAAGCUACACCGGUCGCCACCAUG	hmAG1
Fig. 3A	-	miR-21-5p	miR-21-5p	-	miR-21-5p	GGCCGCUUGAAGUCUUUAAUUAAAUCAACAUCAGUCUGAUAAGCUA<br>UCAACAUCAGUCUGAUAAGCUAGAAGCACUCUGAUUUGACAAUUAU<br>CAACAUCAGUCUGAUAAGCUACACCGGUCGCCACCAUG	hmAG1
Fig. 3A	miR-21-5p	-	miR-21-5p	-	miR-21-5p	GGUCAACAUCAGUCUGAUAAGCUACCGCUUGAAGUCUUUAAUUAAA<br>UCAACAUCAGUCUGAUAAGCUAGAAGCACUCUGAUUUGACAAUUAU<br>CAACAUCAGUCUGAUAAGCUACACCGGUCGCCACCAUG	hmAG1
Fig. 3A	miR-21-5p	miR-21-5p	-	-	miR-21-5p	GGUCAACAUCAGUCUGAUAAGCUAUCAACAUCAGUCUGAUAAGCUA<br>CGAACGGGCACGCUGACAAUUAAGCACUCUGAUUUGACAAUUAUCA<br>ACAUCAGUCUGAUAAGCUACACCGGUCGCCACCAUG	hmAG1
Fig. 3A	-	miR-21-5p	miR-21-5p	miR-21-5p	-	GGCCGCUUGAAGUCUUUAAUUAAAUCAACAUCAGUCUGAUAAGCUA<br>UCAACAUCAGUCUGAUAAGCUAGUCAACAUCAGUCUGAUAAGCUAA<br>AGCACUCUGAUUUGACAAUUACACCGGUCGCCACCAUG	hmAG1
Fig. 3A	miR-21-5p	-	miR-21-5p	miR-21-5p	-	GGUCAACAUCAGUCUGAUAAGCUACCGCUUGAAGUCUUUAAUUAAA<br>UCAACAUCAGUCUGAUAAGCUAGUCAACAUCAGUCUGAUAAGCUAA<br>AGCACUCUGAUUUGACAAUUACACCGGUCGCCACCAUG	hmAG1
Fig. 3A	miR-21-5p	miR-21-5p	-	miR-21-5p	-	GGUCAACAUCAGUCUGAUAAGCUAUCAACAUCAGUCUGAUAAGCUA<br>CGAACGGGCACGCUGACAAUUUCAACAUCAGUCUGAUAAGCUAAAG<br>CACUCUGAUUUGACAAUUACACCGGUCGCCACCAUG	hmAG1
Fig. 3A	-	-	-	-	miR-92a-3p	GGCCGCUUGAAGUCUUUAAUUAAACCCGCUUGAAGUCUUUAAUUAA<br>ACGAACGGGCACGCUGACAAUUAAGCACUCUGAUUUGACAAUUAAC<br>AGGCCGGGACAAGUGCAAUACACCGGUCGCCACCAUG	hmAG1
Fig. 3A	-	-	-	miR-92a-3p	-	GGCCGCUUGAAGUCUUUAAUUAAACCCGCUUGAAGUCUUUAAUUAA<br>ACGAACGGGCACGCUGACAAUUACAGGCCGGGACAAGUGCAAUAAA<br>GCACUCUGAUUUGACAAUUACACCGGUCGCCACCAUG	hmAG1
Fig. 3A	-	-	miR-92a-3p	-	-	GGCCGCUUGAAGUCUUUAAUUAAACCCGCUUGAAGUCUUUAAUUAA<br>AACAGGCCGGGACAAGUGCAAUAAAGCACUCUGAUUUGACAAUUAA<br>AGCACUCUGAUUUGACAAUUACACCGGUCGCCACCAUG	hmAG1
Fig. 3A	-	miR-92a-3p	-	-	-	GGCCGCUUGAAGUCUUUAAUUAAAACAGGCCGGGACAAGUGCAAUA<br>CGAACGGGCACGCUGACAAUUCAAGCACUCUGAUUUGACAAUUACA<br>AGCACUCUGAUUUGACAAUUACACCGGUCGCCACCAUG	hmAG1
Fig. 3A	miR-92a-3p	-	-	-	-	GGACAGGCCGGGACAAGUGCAAUACCGCUUGAAGUCUUUAAUUAAA<br>CGAACGGGCACGCUGACAAUUCAAGCACUCUGAUUUGACAAUUACA<br>AGCACUCUGAUUUGACAAUUACACCGGUCGCCACCAUG	hmAG1

Fig. 3A	-	-	miR-92a-3p	-	-	GGCCGCUUGAAGUCUUUAAUUAAACCGCUUGAAGUCUUUAAUUAAAGACAGGCCGGGACAAGUGCAAUAAAGCACUCUGAUUUGACAAUUAAAGCACUCUGAUUUGACAAUUACACCGGUCGCCACCAUG	hmAG1
Fig. 3A	-	-	-	miR-92a-3p	miR-92a-3p	GGCCGCUUGAAGUCUUUAAUUAAACCCGCUUGAAGUCUUUAAUUAAACGAACGGGCACGCUGACAAUUACAGGCCGGGACAAGUGCAAUAACAGGCCGGGACAAGUGCAAUACACCGGUCGCCACCAUG	hmAG1
Fig. 3A	-	-	miR-92a-3p	-	miR-92a-3p	GGCCGCUUGAAGUCUUUAAUUAAACCGCUUGAAGUCUUUAAUUAAAGACAGGCCGGGACAAGUGCAAUAAAGCACUCUGAUUUGACAAUUAACAGGCCGGGACAAGUGCAAUACACCGGUCGCCACCAUG	hmAG1
Fig. 3A	-	miR-92a-3p	-	-	miR-92a-3p	GGCCGCUUGAAGUCUUUAAUUAAAACAGGCCGGGACAAGUGCAAUACGAACGGGCACGCUGACAAUUAAGCACUCUGAUUUGACAAUUAACAGGCCGGGACAAGUGCAAUACACCGGUCGCCACCAUG	hmAG1
Fig. 3A	miR-92a-3p	-	-	-	miR-92a-3p	GGACAGGCCGGGACAAGUGCAAUACCGCUUGAAGUCUUUAAUUAAACGAACGGGCACGCUGACAAUUAAGCACUCUGAUUUGACAAUUAACAGGCCGGGACAAGUGCAAUACACCGGUCGCCACCAUG	hmAG1
Fig. 3A	-	-	miR-92a-3p	miR-92a-3p	-	GGCCGCUUGAAGUCUUUAAUUAAACCGCUUGAAGUCUUUAAUUAAAGACAGGCCGGGACAAGUGCAAUAACAGGCCGGGACAAGUGCAAUAAAGCACUCUGAUUUGACAAUUACACCGGUCGCCACCAUG	hmAG1
Fig. 3A	-	miR-92a-3p	-	miR-92a-3p	-	GGCCGCUUGAAGUCUUUAAUUAAAACAGGCCGGGACAAGUGCAAUACGAACGGGCACGCUGACAAUUACAGGCCGGGACAAGUGCAAUAAAGCACUCUGAUUUGACAAUUACACCGGUCGCCACCAUG	hmAG1
Fig. 3A	miR-92a-3p	-	-	miR-92a-3p	-	GGACAGGCCGGGACAAGUGCAAUACCGCUUGAAGUCUUUAAUUAAACGAACGGGCACGCUGACAAUUACAGGCCGGGACAAGUGCAAUAAAGCACUCUGAUUUGACAAUUACACCGGUCGCCACCAUG	hmAG1
Fig. 3A	-	miR-92a-3p	miR-92a-3p	-	-	GGCCGCUUGAAGUCUUUAAUUAAAACAGGCCGGGACAAGUGCAAUAGACAGGCCGGGACAAGUGCAAUAAAGCACUCUGAUUUGACAAUUAAAGCACUCUGAUUUGACAAUUACACCGGUCGCCACCAUG	hmAG1
Fig. 3A	miR-92a-3p	-	miR-92a-3p	-	-	GGACAGGCCGGGACAAGUGCAAUACCGCUUGAAGUCUUUAAUUAAAGACAGGCCGGGACAAGUGCAAUAAAGCACUCUGAUUUGACAAUUAAAGCACUCUGAUUUGACAAUUACACCGGUCGCCACCAUG	hmAG1
Fig. 3A	miR-92a-3p	miR-92a-3p	-	-	-	GGACAGGCCGGGACAAGUGCAAUAACAGGCCGGGACAAGUGCAAUACGAACGGGCACGCUGACAAUUCAAGCACUCUGAUUUGACAAUUACAAGCACUCUGAUUUGACAAUUACACCGGUCGCCACCAUG	hmAG1
Fig. 3A	-	miR-92a-3p	-	miR-92a-3p	miR-92a-3p	GGCCGCUUGAAGUCUUUAAUUAAAACAGGCCGGGACAAGUGCAAUACGAACGGGCACGCUGACAAUUACAGGCCGGGACAAGUGCAAUAACAGGCCGGGACAAGUGCAAUACACCGGUCGCCACCAUG	hmAG1
Fig. 3A	miR-92a-3p	-	-	miR-92a-3p	miR-92a-3p	GGACAGGCCGGGACAAGUGCAAUACCGCUUGAAGUCUUUAAUUAAACGAACGGGCACGCUGACAAUUACAGGCCGGGACAAGUGCAAUAACAGGCCGGGACAAGUGCAAUACACCGGUCGCCACCAUG	hmAG1

Fig. 3A	-	miR-92a-3p	miR-92a-3p	-	miR-92a-3p	GGCCGCUUGAAGUCUUUAAUUAAAACAGGCCGGGACAAGUGCAAUA<br>GACAGGCCGGGACAAGUGCAAUAAAGCACUCUGAUUUGACAAUUAA<br>CAGGCCGGGACAAGUGCAAUACACCGGUCGCCACCAUG	hmAG1
Fig. 3A	miR-92a-3p	-	miR-92a-3p	-	miR-92a-3p	GGACAGGCCGGGACAAGUGCAAUACCGCUUGAAGUCUUUAAUUAAA<br>GACAGGCCGGGACAAGUGCAAUAAAGCACUCUGAUUUGACAAUUAA<br>CAGGCCGGGACAAGUGCAAUACACCGGUCGCCACCAUG	hmAG1
Fig. 3A	miR-92a-3p	miR-92a-3p	-	-	miR-92a-3p	GGACAGGCCGGGACAAGUGCAAUAACAGGCCGGGACAAGUGCAAUA<br>CGAACGGGCACGCUGACAAUUAAGCACUCUGAUUUGACAAUUAACA<br>GGCCGGGACAAGUGCAAUACACCGGUCGCCACCAUG	hmAG1
Fig. 3A	-	miR-92a-3p	miR-92a-3p	miR-92a-3p	-	GGCCGCUUGAAGUCUUUAAUUAAAACAGGCCGGGACAAGUGCAAUA<br>GACAGGCCGGGACAAGUGCAAUAACAGGCCGGGACAAGUGCAAUAA<br>AGCACUCUGAUUUGACAAUUACACCGGUCGCCACCAUG	hmAG1
Fig. 3A	miR-92a-3p	-	miR-92a-3p	miR-92a-3p	-	GGACAGGCCGGGACAAGUGCAAUACCGCUUGAAGUCUUUAAUUAAA<br>GACAGGCCGGGACAAGUGCAAUAACAGGCCGGGACAAGUGCAAUAA<br>AGCACUCUGAUUUGACAAUUACACCGGUCGCCACCAUG	hmAG1
Fig. 3A	miR-92a-3p	miR-92a-3p	-	miR-92a-3p	-	GGACAGGCCGGGACAAGUGCAAUAACAGGCCGGGACAAGUGCAAUA<br>CGAACGGGCACGCUGACAAUUACAGGCCGGGACAAGUGCAAUAAAG<br>CACUCUGAUUUGACAAUUACACCGGUCGCCACCAUG	hmAG1
Fig. 4E	miR-4510	miR-199a-5p	-	-	let-7i-5p	GGAACCAUACAUCCUACUCCCUCAGAACAGGUAGUCUGAACACUGG<br>GCGAACGGGCACGCUGACAAUUAAGCACUCUGAUUUGACAAUUAAA<br>CAGCACAAACUACUACCUCACACCGGUCGCCACCAUG	hmAG1
Fig. 4E	miR-135a-5p	miR-135a-5p	miR-302a-5p	miR-135a-5p	miR-135a-5p	GGUCACAUAGGAAUAAAAAGCCAUAUCACAUAGGAAUAAAAAGCCA<br>UAAGCAAGUACAUCCACGUUUAAGUGUCACAUAGGAAUAAAAAGCC<br>AUAUCACAUAGGAAUAAAAAGCCAUACACCGGUCGCCACCAUG	hmKO2
Fig. 4E	miR-302a-5p	miR-199a-5p	miR-199a-5p	miR-302a-5p	miR-199a-5p	GGAGCAAGUACAUCCACGUUUAAGUGAACAGGUAGUCUGAACACUG<br>GGGAACAGGUAGUCUGAACACUGGGACAGCAAGUACAUCCACGUUU<br>AAGUGAACAGGUAGUCUGAACACUGGGCACCGGUCGCCACCAUG	tagBFP
Fig. 4E	miR-200b-3p	miR-200b-3p	miR-200b-3p	miR-200b-3p	miR-200b-3p	GGUCAUCAUUACCAGGCAGUAUUAUCAUCAUUACCAGGCAGUAUUA<br>UCAUCAUUACCAGGCAGUAUUAGAUCAUCAUUACCAGGCAGUAUUA<br>UCAUCAUUACCAGGCAGUAUUACACCGGUCGCCACCAUG	hdKRed
Fig. 5E	-	miR-367-3p	-	-	miR-302a-5p	GGCCGCUUGAAGUCUUUAAUUAAAUCACCAUUGCUAAAGUGCAAUU<br>CGAACGGGCACGCUGACAAUUAAGCACUCUGAUUUGACAAUUAAGC<br>AAGUACAUCCACGUUUAAGUCACCGGUCGCCACCAUG	hmAG1
Fig. 5E	-	miR-27a-3p	-	miR-27a-3p	miR-27a-3p	GGCCGCUUGAAGUCUUUAAUUAAAGCGGAACUUAGCCACUGUGAAA<br>CGAACGGGCACGCUGACAAUUGCGGAACUUAGCCACUGUGAAAGCG<br>GAACUUAGCCACUGUGAAACACCGGUCGCCACCAUG	hmKO2
Fig. 5E	miR-20a-5p	miR-367-3p	miR-373-3p	miR-27a-3p	miR-143-3p	GGCUACCUGCACUAUAAGCACUUUAUCACCAUUGCUAAAGUGCAAU<br>UACACCCCAAAAUCGAAGCACUUCGCGGAACUUAGCCACUGUGAAG<br>AGCUACAGUGCUUCAUCUCACACCGGUCGCCACCAUG	tagBFP

Fig. 5E	miR-17-5p	-	miR-93-5p	miR-17-5p	-	GGCUACCUGCACUGUAAGCACUUUGCCGCUUGAAGUCUUUAAUUAA ACUACCUGCACGAACAGCACUUUGCUACCUGCACUGUAAGCACUUU GAAGCACUCUGAUUUGACAAUUACACCGGUCGCCACCAUG	hdKRed

*[1] The first G is the transcription start site where an anti-reverse cap analog is incorporated. The last AUG is the start codon of the reporter fluorescent protein.

Table S2. 5′UTR sequences of the single-slot mRNAs used in this study.

Transfection	miRNA	miRBase ID	5' UTR sequence*[1,2]	Protein
7	hsa-miR-214-3p	MIMAT0000271	GGUUCCGCGAUCGCGGAUCCACUGCCUGUCUGUGCCUGCUGUAGAUCACACCGGUCGCCACCAUG	hmAG1
8	hsa-miR-127-3p	MIMAT0000446	GGUUCCGCGAUCGCGGAUCCAGCCAAGCUCAGACGGAUCCGAAGAUCACACCGGUCGCCACCAUG	hmAG1
9	hsa-miR-92a-3p	MIMAT0000092	GGUUCCGCGAUCGCGGAUCCACAGGCCGGGACAAGUGCAAUAAGAUCACACCGGUCGCCACCAUG	hmAG1
10	hsa-miR-339-5p	MIMAT0000764	GGUUCCGCGAUCGCGGAUCCCGUGAGCUCCUGGAGGACAGGGAAGAUCCACCGGUCGCCACCAUG	hmAG1
11	hsa-miR-133a	MIMAT0000427	GGUUCCGCGAUCGCGGAUCCCAGCUGGUUGAAGGGGACCAAAAGAUCACACCGGUCGCCACCAUG	hmAG1
12	hsa-miR-17-3p	MIMAT0000071	GGUUCCGCGAUCGCGGAUCCCUACAAGUGCCUUCACUGCAGUAGAUCACACCGGUCGCCACCAUG	hmAG1
13	hsa-miR-1	MIMAT0000416	GGUUCCGCGAUCGCGGAUCCAUACAUACUUCUUUACAUUCCAAGAUCACACCGGUCGCCACCAUG	hmAG1
14	hsa-miR-206	MIMAT0000462	GGUUCCGCGAUCGCGGAUCCCCACACACUUCCUUACAUUCCAAGAUCACACCGGUCGCCACCAUG	hmAG1
15	hsa-miR-197-3p	MIMAT0000227	GGUUCCGCGAUCGCGGAUCCGCUGGGUGGAGAAGGUGGUGAAAGAUCACACCGGUCGCCACCAUG	hmAG1
16	hsa-miR-224-5p	MIMAT0000281	GGUUCCGCGAUCGCGGAUCCAACGGAACCACUAGUGACUUGAGAUCAACACCGGUCGCCACCAUG	hmAG1
17	hsa-miR-365a-3p	MIMAT0000710	GGUUCCGCGAUCGCGGAUCCAUAAGGAUUUUUAGGGGCAUUAAGAUCACACCGGUCGCCACCAUG	hmAG1
18	hsa-miR-183-5p	MIMAT0000261	GGUUCCGCGAUCGCGGAUCCAGUGAAUUCUACCAGUGCCAUAAGAUCACACCGGUCGCCACCAUG	hmAG1
19	hsa-miR-331-3p	MIMAT0000760	GGUUCCGCGAUCGCGGAUCCUUCUAGGAUAGGCCCAGGGGCAGAUCAACACCGGUCGCCACCAUG	hmAG1
20	hsa-miR-203a	MIMAT0000264	GGUUCCGCGAUCGCGGAUCCCUAGUGGUCCUAAACAUUUCACAGAUCACACCGGUCGCCACCAUG	hmAG1
21	hsa-miR-367-3p	MIMAT0000719	GGUUCCGCGAUCGCGGAUCCUCACCAUUGCUAAAGUGCAAUUAGAUCACACCGGUCGCCACCAUG	hmAG1
22	hsa-miR-373-5p	MIMAT0000725	GGUUCCGCGAUCGCGGAUCCGGAAAGCGCCCCCAUUUUGAGUAGAUCACACCGGUCGCCACCAUG	hmAG1
23	hsa-miR-484	MIMAT0002174	GGUUCCGCGAUCGCGGAUCCAUCGGGAGGGGACUGAGCCUGAAGAUCACACCGGUCGCCACCAUG	hmAG1
24	hsa-miR-199a-5p	MIMAT0000231	GGUUCCGCGAUCGCGGAUCCGAACAGGUAGUCUGAACACUGGGAGAUCCACCGGUCGCCACCAUG	hmAG1
25	hsa-miR-370	MIMAT0000722	GGUUCCGCGAUCGCGGAUCCACCAGGUUCCACCCCAGCAGGCAGAUCACACCGGUCGCCACCAUG	hmAG1
26	hsa-miR-382-5p	MIMAT0000737	GGUUCCGCGAUCGCGGAUCCCGAAUCCACCACGAACAACUUCAGAUCACACCGGUCGCCACCAUG	hmAG1
27	hsa-miR-328	MIMAT0000752	GGUUCCGCGAUCGCGGAUCCACGGAAGGGCAGAGAGGGCCAGAGAUCACACCGGUCGCCACCAUG	hmAG1
28	hsa-miR-361-5p	MIMAT0000703	GGUUCCGCGAUCGCGGAUCCGUACCCCUGGAGAUUCUGAUAAAGAUCACACCGGUCGCCACCAUG	hmAG1
29	hsa-miR-486-5p	MIMAT0002177	GGUUCCGCGAUCGCGGAUCCCUCGGGGCAGCUCAGUACAGGAAGAUCACACCGGUCGCCACCAUG	hmAG1
30	hsa-miR-450a-5p	MIMAT0001545	GGUUCCGCGAUCGCGGAUCCAUAUUAGGAACACAUCGCAAAAAGAUCACACCGGUCGCCACCAUG	hmAG1
31	hsa-miR-7-5p	MIMAT0000252	GGUUCCGCGAUCGCGGAUCCACAACAAAAUCACUAGUCUUCCAAGAUCCACCGGUCGCCACCAUG	hmAG1
32	hsa-miR-187-3p	MIMAT0000262	GGUUCCGCGAUCGCGGAUCCCCGGCUGCAACACAAGACACGAAGAUCACACCGGUCGCCACCAUG	hmAG1
33	hsa-miR-505-3p	MIMAT0002876	GGUUCCGCGAUCGCGGAUCCAGGAAACCAGCAAGUGUUGACGAGAUCACACCGGUCGCCACCAUG	hmAG1
34	hsa-miR-182-5p	MIMAT0000259	GGUUCCGCGAUCGCGGAUCCAGUGUGAGUUCUACCAUUGCCAAAGAUCCACCGGUCGCCACCAUG	hmAG1
35	hsa-miR-99a-5p	MIMAT0000097	GGUUCCGCGAUCGCGGAUCCCACAAGAUCGGAUCUACGGGUUAGAUCACACCGGUCGCCACCAUG	hmAG1
36	hsa-miR-100-5p	MIMAT0000098	GGUUCCGCGAUCGCGGAUCCCACAAGUUCGGAUCUACGGGUUAGAUCACACCGGUCGCCACCAUG	hmAG1
37	hsa-let-7a-5p	MIMAT0000062	GGUUCCGCGAUCGCGGAUCCAACUAUACAACCUACUACCUCAAGAUCACACCGGUCGCCACCAUG	hmAG1
38	hsa-miR-191-5p	MIMAT0000440	GGUUCCGCGAUCGCGGAUCCCAGCUGCUUUUGGGAUUCCGUUGAGAUCCACCGGUCGCCACCAUG	hmAG1

39	hsa-miR-19a-3p	MIMAT0000073	GGUUCCGCGAUCGCGGAUCCUCAGUUUUGCAUAGAUUUGCACAAGAUCCACCGGUCGCCACCAUG	hmAG1
40	hsa-miR-99b-5p	MIMAT0000689	GGUUCCGCGAUCGCGGAUCCCGCAAGGUCGGUUCUACGGGUGAGAUCACACCGGUCGCCACCAUG	hmAG1
41	hsa-miR-193b-3p	MIMAT0002819	GGUUCCGCGAUCGCGGAUCCAGCGGGACUUUGAGGGCCAGUUAGAUCACACCGGUCGCCACCAUG	hmAG1
42	hsa-miR-342-3p	MIMAT0000753	GGUUCCGCGAUCGCGGAUCCACGGGUGCGAUUUCUGUGUGAGAAGAUCCACCGGUCGCCACCAUG	hmAG1
43	hsa-miR-320a	MIMAT0000510	GGUUCCGCGAUCGCGGAUCCUCGCCCUCUCAACCCAGCUUUUAGAUCACACCGGUCGCCACCAUG	hmAG1
44	hsa-miR-34a-5p	MIMAT0000255	GGUUCCGCGAUCGCGGAUCCACAACCAGCUAAGACACUGCCAAGAUCACACCGGUCGCCACCAUG	hmAG1
45	hsa-miR-22-3p	MIMAT0000077	GGUUCCGCGAUCGCGGAUCCACAGUUCUUCAACUGGCAGCUUAGAUCACACCGGUCGCCACCAUG	hmAG1
46	hsa-miR-10a-5p	MIMAT0000253	GGUUCCGCGAUCGCGGAUCCCACAAAUUCGGAUCUACAGGGUAAGAUCCACCGGUCGCCACCAUG	hmAG1
47	hsa-miR-423-3p	MIMAT0001340	GGUUCCGCGAUCGCGGAUCCACUGAGGGGCCUCAGACCGAGCUAGAUCCACCGGUCGCCACCAUG	hmAG1
48	hsa-miR-30a-3p	MIMAT0000088	GGUUCCGCGAUCGCGGAUCCGCUGCAAACAUCCGACUGAAAGAGAUCACACCGGUCGCCACCAUG	hmAG1
49	hsa-miR-151a-3p	MIMAT0000757	GGUUCCGCGAUCGCGGAUCCCCUCAAGGAGCUUCAGUCUAGAGAUCAACACCGGUCGCCACCAUG	hmAG1
50	hsa-miR-149-5p	MIMAT0000450	GGUUCCGCGAUCGCGGAUCCGGGAGUGAAGACACGGAGCCAGAAGAUCCACCGGUCGCCACCAUG	hmAG1
51	hsa-miR-296-5p	MIMAT0000690	GGUUCCGCGAUCGCGGAUCCACAGGAUUGAGGGGGGGCCCUAGAUCAACACCGGUCGCCACCAUG	hmAG1
52	hsa-miR-324-3p	MIMAT0000762	GGUUCCGCGAUCGCGGAUCCCCAGCAGCACCUGGGGCAGUAGAUCAAACACCGGUCGCCACCAUG	hmAG1
53	hsa-miR-106b-5p	MIMAT0000680	GGUUCCGCGAUCGCGGAUCCAUCUGCACUGUCAGCACUUUAAGAUCAACACCGGUCGCCACCAUG	hmAG1
54	hsa-miR-129-5p	MIMAT0000242	GGUUCCGCGAUCGCGGAUCCGCAAGCCCAGACCGCAAAAAGAGAUCAACACCGGUCGCCACCAUG	hmAG1
55	hsa-let-7g-5p	MIMAT0000414	GGUUCCGCGAUCGCGGAUCCAACUGUACAAACUACUACCUCAAGAUCACACCGGUCGCCACCAUG	hmAG1
56	hsa-miR-212-3p	MIMAT0000269	GGUUCCGCGAUCGCGGAUCCGGCCGUGACUGGAGACUGUUAAGAUCAACACCGGUCGCCACCAUG	hmAG1
57	hsa-miR-28-5p	MIMAT0000085	GGUUCCGCGAUCGCGGAUCCCUCAAUAGACUGUGAGCUCCUUAGAUCACACCGGUCGCCACCAUG	hmAG1
58	hsa-miR-186-5p	MIMAT0000456	GGUUCCGCGAUCGCGGAUCCAGCCCAAAAGGAGAAUUCUUUGAGAUCACACCGGUCGCCACCAUG	hmAG1
59	hsa-miR-155-5p	MIMAT0000646	GGUUCCGCGAUCGCGGAUCCACCCCUAUCACGAUUAGCAUUAAAGAUCCACCGGUCGCCACCAUG	hmAG1
60	hsa-miR-485-5p	MIMAT0002175	GGUUCCGCGAUCGCGGAUCCGAAUUCAUCACGGCCAGCCUCUAGAUCACACCGGUCGCCACCAUG	hmAG1
61	hsa-miR-330-3p	MIMAT0000751	GGUUCCGCGAUCGCGGAUCCUCUCUGCAGGCCGUGUGCUUUGCAGAUCCACCGGUCGCCACCAUG	hmAG1
62	hsa-miR-345-5p	MIMAT0000772	GGUUCCGCGAUCGCGGAUCCGAGCCCUGGACUAGGAGUCAGCAGAUCACACCGGUCGCCACCAUG	hmAG1
63	hsa-miR-500a-3p	MIMAT0002871	GGUUCCGCGAUCGCGGAUCCCAGAAUCCUUGCCCAGGUGCAUAGAUCACACCGGUCGCCACCAUG	hmAG1
64	hsa-miR-192-5p	MIMAT0000222	GGUUCCGCGAUCGCGGAUCCGGCUGUCAAUUCAUAGGUCAGAGAUCAACACCGGUCGCCACCAUG	hmAG1
65	hsa-miR-335-5p	MIMAT0000765	GGUUCCGCGAUCGCGGAUCCACAUUUUUCGUUAUUGCUCUUGAAGAUCCACCGGUCGCCACCAUG	hmAG1
66	hsa-miR-140-5p	MIMAT0000431	GGUUCCGCGAUCGCGGAUCCCUACCAUAGGGUAAAACCACUGAGAUCACACCGGUCGCCACCAUG	hmAG1
67	hsa-miR-18a-3p	MIMAT0002891	GGUUCCGCGAUCGCGGAUCCCCAGAAGGAGCACUUAGGGCAGUAGAUCCACCGGUCGCCACCAUG	hmAG1
68	hsa-miR-148a-3p	MIMAT0000243	GGUUCCGCGAUCGCGGAUCCACAAAGUUCUGUAGUGCACUGAAGAUCACACCGGUCGCCACCAUG	hmAG1
69	hsa-miR-124-3p	MIMAT0000422	GGUUCCGCGAUCGCGGAUCCGGCAUUCACCGCGUGCCUUAAGAUCAAACACCGGUCGCCACCAUG	hmAG1
70	hsa-miR-9-5p	MIMAT0000441	GGUUCCGCGAUCGCGGAUCCUCAUACAGCUAGAUAACCAAAGAAGAUCCACCGGUCGCCACCAUG	hmAG1
71	hsa-miR-126-3p	MIMAT0000445	GGUUCCGCGAUCGCGGAUCCCGCAUUAUUACUCACGGUACGAAGAUCACACCGGUCGCCACCAUG	hmAG1
72	hsa-miR-101-3p	MIMAT0000099	GGUUCCGCGAUCGCGGAUCCUUCAGUUAUCACAGUACUGUAAGAUCAACACCGGUCGCCACCAUG	hmAG1
73	hsa-miR-135a-5p	MIMAT0000428	GGUUCCGCGAUCGCGGAUCCUCACAUAGGAAUAAAAAGCCAUAAGAUCCACCGGUCGCCACCAUG	hmAG1
74	hsa-miR-141-3p	MIMAT0000432	GGUUCCGCGAUCGCGGAUCCCCAUCUUUACCAGACAGUGUUAAGAUCACACCGGUCGCCACCAUG	hmAG1

75	hsa-miR-154-5p	MIMAT0000452	GGUUCCGCGAUCGCGGAUCCCGAAGGCAACACGGAUAACCUAAGAUCACACCGGUCGCCACCAUG	hmAG1
76	hsa-miR-195-5p	MIMAT0000461	GGUUCCGCGAUCGCGGAUCCGCCAAUAUUUCUGUGCUGCUAAGAUCAACACCGGUCGCCACCAUG	hmAG1
77	hsa-miR-200b-3p	MIMAT0000318	GGUUCCGCGAUCGCGGAUCCUCAUCAUUACCAGGCAGUAUUAAGAUCACACCGGUCGCCACCAUG	hmAG1
78	hsa-miR-200c-3p	MIMAT0000617	GGUUCCGCGAUCGCGGAUCCUCCAUCAUUACCCGGCAGUAUUAAGAUCCACCGGUCGCCACCAUG	hmAG1
79	hsa-miR-210	MIMAT0000267	GGUUCCGCGAUCGCGGAUCCUCAGCCGCUGUCACACGCACAGAGAUCACACCGGUCGCCACCAUG	hmAG1
80	hsa-miR-302a-5p	MIMAT0000683	GGUUCCGCGAUCGCGGAUCCAGCAAGUACAUCCACGUUUAAGUAGAUCCACCGGUCGCCACCAUG	hmAG1
81	hsa-miR-375	MIMAT0000728	GGUUCCGCGAUCGCGGAUCCUCACGCGAGCCGAACGAACAAAAGAUCACACCGGUCGCCACCAUG	hmAG1
82	hsa-miR-378a-3p	MIMAT0000732	GGUUCCGCGAUCGCGGAUCCCCUUCUGACUCCAAGUCCAGUAGAUCAACACCGGUCGCCACCAUG	hmAG1
83	hsa-miR-512-5p	MIMAT0002822	GGUUCCGCGAUCGCGGAUCCGAAAGUGCCCUCAAGGCUGAGUGAGAUCCACCGGUCGCCACCAUG	hmAG1
84	hsa-miR-518b	MIMAT0002844	GGUUCCGCGAUCGCGGAUCCACCUCUAAAGGGGAGCGCUUUGAGAUCACACCGGUCGCCACCAUG	hmAG1
85	hsa-miR-518c-3p	MIMAT0002848	GGUUCCGCGAUCGCGGAUCCACACUCUAAAGAGAAGCGCUUUGAGAUCCACCGGUCGCCACCAUG	hmAG1
86	hsa-miR-519d	MIMAT0002853	GGUUCCGCGAUCGCGGAUCCCACUCUAAAGGGAGGCACUUUGAGAUCACACCGGUCGCCACCAUG	hmAG1
87	hsa-miR-520g	MIMAT0002858	GGUUCCGCGAUCGCGGAUCCACACUCUAAAGGGAAGCACUUUGUAGAUCACCGGUCGCCACCAUG	hmAG1
88	hsa-miR-523-3p	MIMAT0002840	GGUUCCGCGAUCGCGGAUCCACCCUCUAUAGGGAAGCGCGUUCAGAUCCACCGGUCGCCACCAUG	hmAG1
89	hsa-miR-525-5p	MIMAT0002838	GGUUCCGCGAUCGCGGAUCCAGAAAGUGCAUCCCUCUGGAGAGAUCAACACCGGUCGCCACCAUG	hmAG1
90	hsa-miR-526a	MIMAT0002845	GGUUCCGCGAUCGCGGAUCCCAGAAAGUGCUUCCCUCUAGAGAGAUCACACCGGUCGCCACCAUG	hmAG1
91	hsa-miR-362-3p	MIMAT0004683	GGUUCCGCGAUCGCGGAUCCUGAAUCCUUGAAUAGGUGUGUUAGAUCACACCGGUCGCCACCAUG	hmAG1
92	hsa-miR-589-5p	MIMAT0004799	GGUUCCGCGAUCGCGGAUCCCUCAGAGCAGACGUGGUUCUCAAGAUCACACCGGUCGCCACCAUG	hmAG1
93	hsa-miR-671-5p	MIMAT0003880	GGUUCCGCGAUCGCGGAUCCCUCCAGCCCCUCCAGGGCUUCCUAGAUCCACCGGUCGCCACCAUG	hmAG1
94	hsa-miR-506-3p	MIMAT0002878	GGUUCCGCGAUCGCGGAUCCUCUACUCAGAAGGGUGCCUUAAGAUCAACACCGGUCGCCACCAUG	hmAG1
95	hsa-miR-125b-1-3p	MIMAT0004592	GGUUCCGCGAUCGCGGAUCCAGCUCCCAAGAGCCUAACCCGUAGAUCACACCGGUCGCCACCAUG	hmAG1
96	hsa-miR-30b-3p	MIMAT0004589	GGUUCCGCGAUCGCGGAUCCGAAGUAAACAUCCACCUCCCAGAGAUCACACCGGUCGCCACCAUG	hmAG1
7	hsa-miR-26a-5p	MIMAT0000082	GGUUCCGCGAUCGCGGAUCCAGCCUAUCCUGGAUUACUUGAAAGAUCACACCGGUCGCCACCAUG	tagBFP
8	hsa-miR-24-3p	MIMAT0000080	GGUUCCGCGAUCGCGGAUCCCUGUUCCUGCUGAACUGAGCCAAGAUCACACCGGUCGCCACCAUG	tagBFP
9	hsa-miR-27a-3p	MIMAT0000084	GGUUCCGCGAUCGCGGAUCCGCGGAACUUAGCCACUGUGAAAGAUCAACACCGGUCGCCACCAUG	tagBFP
10	hsa-miR-21-5p	MIMAT0000076	GGUUCCGCGAUCGCGGAUCCUCAACAUCAGUCUGAUAAGCUAAGAUCACACCGGUCGCCACCAUG	tagBFP
11	hsa-miR-143-3p	MIMAT0000435	GGUUCCGCGAUCGCGGAUCCGAGCUACAGUGCUUCAUCUCAAGAUCAACACCGGUCGCCACCAUG	tagBFP
12	hsa-let-7i-5p	MIMAT0000415	GGUUCCGCGAUCGCGGAUCCAACAGCACAAACUACUACCUCAAGAUCACACCGGUCGCCACCAUG	tagBFP
13	hsa-miR-409-3p	MIMAT0001639	GGUUCCGCGAUCGCGGAUCCAGGGGUUCACCGAGCAACAUUCAGAUCACACCGGUCGCCACCAUG	tagBFP
14	hsa-miR-196b-5p	MIMAT0001080	GGUUCCGCGAUCGCGGAUCCCCCAACAACAGGAAACUACCUAAGAUCACACCGGUCGCCACCAUG	tagBFP
15	hsa-miR-501-5p	MIMAT0002872	GGUUCCGCGAUCGCGGAUCCUCUCACCCAGGGACAAAGGAUUAGAUCACACCGGUCGCCACCAUG	tagBFP
16	hsa-miR-362-5p	MIMAT0000705	GGUUCCGCGAUCGCGGAUCCACUCACACCUAGGUUCCAAGGAUUAGAUCACCGGUCGCCACCAUG	tagBFP
17	hsa-miR-193a-3p	MIMAT0000459	GGUUCCGCGAUCGCGGAUCCACUGGGACUUUGUAGGCCAGUUAGAUCACACCGGUCGCCACCAUG	tagBFP
18	hsa-miR-134	MIMAT0000447	GGUUCCGCGAUCGCGGAUCCCCCCUCUGGUCAACCAGUCACAAGAUCACACCGGUCGCCACCAUG	tagBFP
19	hsa-miR-299-3p	MIMAT0000687	GGUUCCGCGAUCGCGGAUCCAAGCGGUUUACCAUCCCACAUAAGAUCACACCGGUCGCCACCAUG	tagBFP
20	hsa-miR-483-3p	MIMAT0002173	GGUUCCGCGAUCGCGGAUCCAAGACGGGAGGAGAGGAGUGAAGAUCAACACCGGUCGCCACCAUG	tagBFP

21	hsa-miR-150-5p	MIMAT0000451	GGUUCCGCGAUCGCGGAUCCCACUGGUACAAGGGUUGGGAGAAGAUCACACCGGUCGCCACCAUG	tagBFP
22	hsa-miR-200a-3p	MIMAT0000682	GGUUCCGCGAUCGCGGAUCCACAUCGUUACCAGACAGUGUUAAGAUCACACCGGUCGCCACCAUG	tagBFP
23	hsa-miR-133b	MIMAT0000770	GGUUCCGCGAUCGCGGAUCCUAGCUGGUUGAAGGGGACCAAAAGAUCACACCGGUCGCCACCAUG	tagBFP
24	hsa-miR-520c-3p	MIMAT0002846	GGUUCCGCGAUCGCGGAUCCACCCUCUAAAAGGAAGCACUUUAGAUCACACCGGUCGCCACCAUG	tagBFP
25	hsa-miR-378a-5p	MIMAT0000731	GGUUCCGCGAUCGCGGAUCCACACAGGACCUGGAGUCAGGAGAGAUCACACCGGUCGCCACCAUG	tagBFP
26	hsa-miR-373-3p	MIMAT0000726	GGUUCCGCGAUCGCGGAUCCACACCCCAAAAUCGAAGCACUUCAGAUCCACCGGUCGCCACCAUG	tagBFP
27	hsa-miR-492	MIMAT0002812	GGUUCCGCGAUCGCGGAUCCAAGAAUCUUGUCCCGCAGGUCCUAGAUCCACCGGUCGCCACCAUG	tagBFP
28	hsa-miR-509-3p	MIMAT0002881	GGUUCCGCGAUCGCGGAUCCCUACCCACAGACGUACCAAUCAAGAUCACACCGGUCGCCACCAUG	tagBFP
29	hsa-miR-516b-5p	MIMAT0002859	GGUUCCGCGAUCGCGGAUCCAAAGUGCUUCUUACCUCCAGAUAGAUCACACCGGUCGCCACCAUG	tagBFP
30	hsa-miR-518c-5p	MIMAT0002847	GGUUCCGCGAUCGCGGAUCCCAGAAAGUGCUUCCCUCCAGAGAAGAUCCACCGGUCGCCACCAUG	tagBFP
31	hsa-miR-520f	MIMAT0002830	GGUUCCGCGAUCGCGGAUCCAACCCUCUAAAAGGAAGCACUUAGAUCACACCGGUCGCCACCAUG	tagBFP
32	hsa-miR-98-5p	MIMAT0000096	GGUUCCGCGAUCGCGGAUCCAACAAUACAACUUACUACCUCAAGAUCACACCGGUCGCCACCAUG	tagBFP
33	hsa-miR-320b	MIMAT0005792	GGUUCCGCGAUCGCGGAUCCUUGCCCUCUCAACCCAGCUUUUAGAUCACACCGGUCGCCACCAUG	tagBFP
34	hsa-miR-423-5p	MIMAT0004748	GGUUCCGCGAUCGCGGAUCCAAAGUCUCGCUCUCUGCCCCUCAAGAUCCACCGGUCGCCACCAUG	tagBFP
35	hsa-miR-140-3p	MIMAT0004597	GGUUCCGCGAUCGCGGAUCCCCGUGGUUCUACCCUGUGGUAAGAUCAACACCGGUCGCCACCAUG	tagBFP
36	hsa-miR-378c	MIMAT0016847	GGUUCCGCGAUCGCGGAUCCCCACUCUUCUGACUCCAAGUCCAGUAUCCACCGGUCGCCACCAUG	tagBFP
37	hsa-miR-185-5p	MIMAT0000455	GGUUCCGCGAUCGCGGAUCCUCAGGAACUGCCUUUCUCUCCAAGAUCACACCGGUCGCCACCAUG	tagBFP
38	hsa-miR-378b	MIMAT0014999	GGUUCCGCGAUCGCGGAUCCUUCUGCCUCCAAGUCCAGUAGAUCCAAACACCGGUCGCCACCAUG	tagBFP
39	hsa-miR-128	MIMAT0000424	GGUUCCGCGAUCGCGGAUCCAAAGAGACCGGUUCACUGUGAAGAUCAACACCGGUCGCCACCAUG	tagBFP
40	hsa-miR-509-5p	MIMAT0004779	GGUUCCGCGAUCGCGGAUCCUGAUUGCCACUGUCUGCAGUAAGAUCAACACCGGUCGCCACCAUG	tagBFP
41	hsa-miR-340-5p	MIMAT0004692	GGUUCCGCGAUCGCGGAUCCAAUCAGUCUCAUUGCUUUAUAAAGAUCACACCGGUCGCCACCAUG	tagBFP
42	hsa-miR-25-5p	MIMAT0004498	GGUUCCGCGAUCGCGGAUCCCAAUUGCCCAAGUCUCCGCCUAGAUCAACACCGGUCGCCACCAUG	tagBFP
43	hsa-miR-92b-3p	MIMAT0003218	GGUUCCGCGAUCGCGGAUCCGGAGGCCGGGACGAGUGCAAUAAGAUCACACCGGUCGCCACCAUG	tagBFP
44	hsa-miR-320c	MIMAT0005793	GGUUCCGCGAUCGCGGAUCCACCCUCUCAACCCAGCUUUUAGAUCAAACACCGGUCGCCACCAUG	tagBFP
45	hsa-miR-508-3p	MIMAT0002880	GGUUCCGCGAUCGCGGAUCCUCUACUCCAAAAGGCUACAAUCAAGAUCCACCGGUCGCCACCAUG	tagBFP
46	hsa-miR-744-5p	MIMAT0004945	GGUUCCGCGAUCGCGGAUCCUGCUGUUAGCCCUAGCCCCGCAAGAUCACACCGGUCGCCACCAUG	tagBFP
47	hsa-miR-3180-3p	MIMAT0015058	GGUUCCGCGAUCGCGGAUCCGGCCUCCGGAAGCUCCGCCCCAAGAUCACACCGGUCGCCACCAUG	tagBFP
48	hsa-miR-30d-3p	MIMAT0004551	GGUUCCGCGAUCGCGGAUCCGCAGCAAACAUCUGACUGAAAGAGAUCACACCGGUCGCCACCAUG	tagBFP
49	hsa-miR-215	MIMAT0000272	GGUUCCGCGAUCGCGGAUCCGUCUGUCAAUUCAUAGGUCAUAGAUCAACACCGGUCGCCACCAUG	tagBFP
50	hsa-miR-4510	MIMAT0019047	GGUUCCGCGAUCGCGGAUCCAACCAUACAUCCUACUCCCUCAAGAUCACACCGGUCGCCACCAUG	tagBFP
51	hsa-miR-1260a	MIMAT0005911	GGUUCCGCGAUCGCGGAUCCUGGUGGCAGAGGUGGGAUAGAUCACCGGUCGCCACCAUG	tagBFP
52	hsa-miR-151a-5p	MIMAT0004697	GGUUCCGCGAUCGCGGAUCCACUAGACUGUGAGCUCCUCGAAGAUCAACACCGGUCGCCACCAUG	tagBFP
53	hsa-miR-584-5p	MIMAT0003249	GGUUCCGCGAUCGCGGAUCCCUCAGUCCCAGGCAAACCAUAAAGAUCACACCGGUCGCCACCAUG	tagBFP
54	hsa-miR-503-5p	MIMAT0002874	GGUUCCGCGAUCGCGGAUCCCUGCAGAACUGUUCCCGCUGCUAAGAUCCACCGGUCGCCACCAUG	tagBFP
55	hsa-miR-34c-5p	MIMAT0000686	GGUUCCGCGAUCGCGGAUCCGCAAUCAGCUAACUACACUGCCUAGAUCCACCGGUCGCCACCAUG	tagBFP
56	hsa-miR-3180	MIMAT0018178	GGUUCCGCGAUCGCGGAUCCCUCCGGAAGCUCCGCCCCAAGAUCCAAACACCGGUCGCCACCAUG	tagBFP

57	hsa-miR-514a-3p	MIMAT0002883	GGUUCCGCGAUCGCGGAUCCUCUACUCACAGAAGUGUCAAUAGAUCAACACCGGUCGCCACCAUG	tagBFP
58	hsa-miR-374b-5p	MIMAT0004955	GGUUCCGCGAUCGCGGAUCCCACUUAGCAGGUUGUAUUAUAUAGAUCACACCGGUCGCCACCAUG	tagBFP
59	hsa-miR-28-3p	MIMAT0004502	GGUUCCGCGAUCGCGGAUCCUCCAGGAGCUCACAAUCUAGUGAGAUCACACCGGUCGCCACCAUG	tagBFP
60	hsa-miR-1307-5p	MIMAT0022727	GGUUCCGCGAUCGCGGAUCCAGCCGGUCGAGGUCCGGUCGAAGAUCAACACCGGUCGCCACCAUG	tagBFP
61	hsa-miR-21-3p	MIMAT0004494	GGUUCCGCGAUCGCGGAUCCACAGCCCAUCGACUGGUGUUGAGAUCAACACCGGUCGCCACCAUG	tagBFP
62	hsa-miR-193a-5p	MIMAT0004614	GGUUCCGCGAUCGCGGAUCCUCAUCUCGCCCGCAAAGACCCAAGAUCACACCGGUCGCCACCAUG	tagBFP
63	hsa-miR-1269a	MIMAT0005923	GGUUCCGCGAUCGCGGAUCCCCAGUAGCACGGCUCAGUCCAGAGAUCACACCGGUCGCCACCAUG	tagBFP
64	hsa-miR-365b-3p	MIMAT0022834	GGUUCCGCGAUCGCGGAUCCAUAAGGAUUUUUAGGGGCAUUAAGAUCACACCGGUCGCCACCAUG	tagBFP
65	hsa-miR-4286	MIMAT0016916	GGUUCCGCGAUCGCGGAUCCGGUACCAGGAGUGGGGUAGAUCCACCGGUCGCCACCAUG	tagBFP
66	hsa-miR-4454	MIMAT0018976	GGUUCCGCGAUCGCGGAUCCUGGUGCCGUGACUCGGAUCCAGAUCAAACACCGGUCGCCACCAUG	tagBFP
67	hsa-miR-138-5p	MIMAT0000430	GGUUCCGCGAUCGCGGAUCCCGGCCUGAUUCACAACACCAGCUAGAUCCACCGGUCGCCACCAUG	tagBFP
68	hsa-miR-1307-3p	MIMAT0005951	GGUUCCGCGAUCGCGGAUCCCACGACCGACGCCACGCCGAGUAGAUCACACCGGUCGCCACCAUG	tagBFP
69	hsa-miR-652-3p	MIMAT0003322	GGUUCCGCGAUCGCGGAUCCCACAACCCUAGUGGCGCCAUUAGAUCAACACCGGUCGCCACCAUG	tagBFP
70	hsa-miR-502-3p	MIMAT0004775	GGUUCCGCGAUCGCGGAUCCUGAAUCCUUGCCCAGGUGCAUUAGAUCACACCGGUCGCCACCAUG	tagBFP
71	hsa-miR-92b-5p	MIMAT0004792	GGUUCCGCGAUCGCGGAUCCCACUGCACCGCGUCCCGUCCCUAGAUCACACCGGUCGCCACCAUG	tagBFP
72	hsa-miR-501-3p	MIMAT0004774	GGUUCCGCGAUCGCGGAUCCAGAAUCCUUGCCCGGGUGCAUUAGAUCACACCGGUCGCCACCAUG	tagBFP
73	hsa-miR-1285-3p	MIMAT0005876	GGUUCCGCGAUCGCGGAUCCAGGUCUCACUUUGUUGCCCAGAAGAUCACACCGGUCGCCACCAUG	tagBFP
74	hsa-miR-296-3p	MIMAT0004679	GGUUCCGCGAUCGCGGAUCCGGAGAGCCUCCACCCAACCCUCAGAUCACACCGGUCGCCACCAUG	tagBFP
75	hsa-miR-506-5p	MIMAT0022701	GGUUCCGCGAUCGCGGAUCCUUAAGUAACACCUUCCUGAAUAAGAUCACACCGGUCGCCACCAUG	tagBFP
76	hsa-miR-361-3p	MIMAT0004682	GGUUCCGCGAUCGCGGAUCCAAAUCAGAAUCACACCUGGGGGAAGAUCCACCGGUCGCCACCAUG	tagBFP
77	hsa-miR-582-3p	MIMAT0004797	GGUUCCGCGAUCGCGGAUCCGGUUCAGUUGUUCAACCAGUUAAGAUCACACCGGUCGCCACCAUG	tagBFP
78	hsa-miR-422a	MIMAT0001339	GGUUCCGCGAUCGCGGAUCCGCCUUCUGACCCUAAGUCCAGUAGAUCACACCGGUCGCCACCAUG	tagBFP
79	hsa-miR-98-3p	MIMAT0022842	GGUUCCGCGAUCGCGGAUCCGGGAAAGUAGUAAGUUGUAUAGAGAUCACACCGGUCGCCACCAUG	tagBFP
80	hsa-miR-499a-5p	MIMAT0002870	GGUUCCGCGAUCGCGGAUCCAAACAUCACUGCAAGUCUUAAAGAUCAACACCGGUCGCCACCAUG	tagBFP
81	hsa-miR-24-2-5p	MIMAT0004497	GGUUCCGCGAUCGCGGAUCCCUGUGUUUCAGCUCAGUAGGCAAGAUCACACCGGUCGCCACCAUG	tagBFP
82	hsa-miR-105-5p	MIMAT0000102	GGUUCCGCGAUCGCGGAUCCACCACAGGAGUCUGAGCAUUUGAAGAUCCACCGGUCGCCACCAUG	tagBFP
83	hsa-miR-381-3p	MIMAT0000736	GGUUCCGCGAUCGCGGAUCCACAGAGAGCUUGCCCUUGUAUAAGAUCACACCGGUCGCCACCAUG	tagBFP
84	hsa-miR-181b-3p	MIMAT0022692	GGUUCCGCGAUCGCGGAUCCUUGCAUUCAUUGUUCAGUGAGAGAUCAACACCGGUCGCCACCAUG	tagBFP
85	hsa-miR-122-5p	MIMAT0000421	GGUUCCGCGAUCGCGGAUCCCAAACACCAUUGUCACACUCCAAGAUCACACCGGUCGCCACCAUG	tagBFP
86	hsa-miR-27a-5p	MIMAT0004501	GGUUCCGCGAUCGCGGAUCCUGCUCACAAGCAGCUAAGCCCUAGAUCACACCGGUCGCCACCAUG	tagBFP
87	hsa-miR-505-5p	MIMAT0004776	GGUUCCGCGAUCGCGGAUCCACAUCAAUACUUCCUGGCUCCCAGAUCACACCGGUCGCCACCAUG	tagBFP
88	hsa-miR-454-3p	MIMAT0003885	GGUUCCGCGAUCGCGGAUCCACCCUAUAAGCAAUAUUGCACUAAGAUCCACCGGUCGCCACCAUG	tagBFP
89	hsa-miR-301a-3p	MIMAT0000688	GGUUCCGCGAUCGCGGAUCCGCUUUGACAAUACUAUUGCACUGAGAUCCACCGGUCGCCACCAUG	tagBFP
90	hsa-miR-510	MIMAT0002882	GGUUCCGCGAUCGCGGAUCCGUGAUUGCCACUCUCCUGAGUAAGAUCACACCGGUCGCCACCAUG	tagBFP
91	hsa-miR-411-5p	MIMAT0003329	GGUUCCGCGAUCGCGGAUCCCGUACGCUAUACGGUCUACUAAGAUCAACACCGGUCGCCACCAUG	tagBFP
92	hsa-miR-29c-5p	MIMAT0004673	GGUUCCGCGAUCGCGGAUCCGAACACCAGGAGAAAUCGGUCAAGAUCACACCGGUCGCCACCAUG	tagBFP

93	hsa-miR-148b-5p	MIMAT0004699	GGUUCCGCGAUCGCGGAUCCGCCUGAGUGUAUAACAGAACUUAGAUCACACCGGUCGCCACCAUG	tagBFP
94	hsa-miR-204-3p	MIMAT0022693	GGUUCCGCGAUCGCGGAUCCACGUCCCUUUGCCUUCCCAGCAGAUCAACACCGGUCGCCACCAUG	tagBFP
95	hsa-miR-486-3p	MIMAT0004762	GGUUCCGCGAUCGCGGAUCCAUCCUGUACUGAGCUGCCCCGAGAUCAACACCGGUCGCCACCAUG	tagBFP
96	hsa-miR-125b-2-3p	MIMAT0004603	GGUUCCGCGAUCGCGGAUCCGUCCCAAGAGCCUGACUUGUGAAGAUCACACCGGUCGCCACCAUG	tagBFP
7	hsa-miR-16-5p	MIMAT0000069	GGUUCCGCGAUCGCGGAUCCCGCCAAUAUUUACGUGCUGCUAAGAUCACACCGGUCGCCACCAUG	hdKRed
8	hsa-miR-17-5p	MIMAT0000070	GGUUCCGCGAUCGCGGAUCCCUACCUGCACUGUAAGCACUUUGAGAUCCACCGGUCGCCACCAUG	hdKRed
9	hsa-miR-125b-5p	MIMAT0000423	GGUUCCGCGAUCGCGGAUCCUCACAAGUUAGGGUCUCAGGGAAGAUCACACCGGUCGCCACCAUG	hdKRed
10	hsa-miR-93-5p	MIMAT0000093	GGUUCCGCGAUCGCGGAUCCCUACCUGCACGAACAGCACUUUGAGAUCCACCGGUCGCCACCAUG	hdKRed
11	hsa-miR-20a-5p	MIMAT0000075	GGUUCCGCGAUCGCGGAUCCCUACCUGCACUAUAAGCACUUUAAGAUCCACCGGUCGCCACCAUG	hdKRed
12	hsa-miR-106a-5p	MIMAT0000103	GGUUCCGCGAUCGCGGAUCCCUACCUGCACUGUAAGCACUUUUAGAUCCACCGGUCGCCACCAUG	hdKRed
13	hsa-miR-145-5p	MIMAT0000437	GGUUCCGCGAUCGCGGAUCCAGGGAUUCCUGGGAAAACUGGACAGAUCCACCGGUCGCCACCAUG	hdKRed
14	hsa-miR-4531	MIMAT0019070	GGUUCCGCGAUCGCGGAUCCUCAGAAGCCUUCUCCAUAGAUCCACCGGUCGCCACCAUG	hdKRed
15	hsa-miR-193b-5p	MIMAT0004767	GGUUCCGCGAUCGCGGAUCCUCAUCUCGCCCUCAAAACCCCGAGAUCACACCGGUCGCCACCAUG	hdKRed
16	hsa-miR-181a-2-3p	MIMAT0004558	GGUUCCGCGAUCGCGGAUCCGGUACAGUCAACGGUCAGUGGUAGAUCACACCGGUCGCCACCAUG	hdKRed
17	hsa-miR-1301	MIMAT0005797	GGUUCCGCGAUCGCGGAUCCGAAGUCACUCCCAGGCAGCUGCAAGAUCCACCGGUCGCCACCAUG	hdKRed
18	hsa-miR-877-5p	MIMAT0004949	GGUUCCGCGAUCGCGGAUCCCCCUGCGCCAUCUCCUCUACAGAUCAAACACCGGUCGCCACCAUG	hdKRed
19	hsa-miR-4443	MIMAT0018961	GGUUCCGCGAUCGCGGAUCCAAAACCCACGCCUCCAAAGAUCCACCGGUCGCCACCAUG	hdKRed
20	hsa-miR-425-5p	MIMAT0003393	GGUUCCGCGAUCGCGGAUCCUCAACGGGAGUGAUCGUGUCAUUAGAUCCACCGGUCGCCACCAUG	hdKRed
21	hsa-miR-320d	MIMAT0006764	GGUUCCGCGAUCGCGGAUCCUCCUCUCAACCCAGCUUUUAGAUCCAAACACCGGUCGCCACCAUG	hdKRed
22	hsa-let-7a-3p	MIMAT0004481	GGUUCCGCGAUCGCGGAUCCGAAAGACAGUAGAUUGUAUAGAGAUCAACACCGGUCGCCACCAUG	hdKRed
23	hsa-miR-92a-1-5p	MIMAT0004507	GGUUCCGCGAUCGCGGAUCCAGCAUUGCAACCGAUCCCAACCUAGAUCCACCGGUCGCCACCAUG	hdKRed
24	hsa-miR-365b-5p	MIMAT0022833	GGUUCCGCGAUCGCGGAUCCACAGCUGCCCCUGAAAGUCCCUAGAUCACACCGGUCGCCACCAUG	hdKRed
25	hsa-miR-142-3p	MIMAT0000434	GGUUCCGCGAUCGCGGAUCCUCCAUAAAGUAGGAAACACUACAAGAUCCACCGGUCGCCACCAUG	hdKRed
26	hsa-miR-320e	MIMAT0015072	GGUUCCGCGAUCGCGGAUCCCCUUCUCAACCCAGCUUUAGAUCACCGGUCGCCACCAUG	hdKRed
27	hsa-miR-106b-3p	MIMAT0004672	GGUUCCGCGAUCGCGGAUCCGCAGCAAGUACCCACAGUGCGGAGAUCACACCGGUCGCCACCAUG	hdKRed
28	hsa-miR-548i	MIMAT0005935	GGUUCCGCGAUCGCGGAUCCGGCAAAAUCCGCAAUUACUUUUAGAUCACACCGGUCGCCACCAUG	hdKRed
29	hsa-miR-1261	MIMAT0005913	GGUUCCGCGAUCGCGGAUCCAAGCCAAAGCCUUAUCCAUAGAUCCAAACACCGGUCGCCACCAUG	hdKRed
30	hsa-miR-9-3p	MIMAT0000442	GGUUCCGCGAUCGCGGAUCCACUUUCGGUUAUCUAGCUUUAUAGAUCACACCGGUCGCCACCAUG	hdKRed
31	hsa-miR-129-1-3p	MIMAT0004548	GGUUCCGCGAUCGCGGAUCCAUACUUUUUGGGGUAAGGGCUUAGAUCACACCGGUCGCCACCAUG	hdKRed
32	hsa-miR-424-3p	MIMAT0004749	GGUUCCGCGAUCGCGGAUCCAUAGCAGCGCCUCACGUUUUGAGAUCAACACCGGUCGCCACCAUG	hdKRed
33	hsa-miR-760	MIMAT0004957	GGUUCCGCGAUCGCGGAUCCUCCCCACAGACCCAGAGCCGAGAUCAAACACCGGUCGCCACCAUG	hdKRed
34	hsa-miR-365a-5p	MIMAT0009199	GGUUCCGCGAUCGCGGAUCCCACAUCUGCCCCCAAAAGUCCCUAGAUCCACCGGUCGCCACCAUG	hdKRed
35	hsa-miR-374a-5p	MIMAT0000727	GGUUCCGCGAUCGCGGAUCCCACUUAUCAGGUUGUAUUAUAAAGAUCACACCGGUCGCCACCAUG	hdKRed
36	hsa-miR-873-5p	MIMAT0004953	GGUUCCGCGAUCGCGGAUCCAGGAGACUCACAAGUUCCUGCAGAUCAACACCGGUCGCCACCAUG	hdKRed
37	hsa-miR-30c-2-3p	MIMAT0004550	GGUUCCGCGAUCGCGGAUCCAGAGUAAACAGCCUUCUCCCAGAGAUCACACCGGUCGCCACCAUG	hdKRed
38	hsa-miR-99b-3p	MIMAT0004678	GGUUCCGCGAUCGCGGAUCCCGGACCCACAGACACGAGCUUGAGAUCACACCGGUCGCCACCAUG	hdKRed

39	hsa-miR-4532	MIMAT0019071	GGUUCCGCGAUCGCGGAUCCCGCCGGGCUCCCCGGGGAGAUCCACCGGUCGCCACCAUG	hdKRed
40	hsa-miR-20b-3p	MIMAT0004752	GGUUCCGCGAUCGCGGAUCCCUGGAAGUGCCCAUACUACAGUAGAUCACACCGGUCGCCACCAUG	hdKRed
41	hsa-miR-4448	MIMAT0018967	GGUUCCGCGAUCGCGGAUCCUACCCCUAGACCAAGGAGCCAGAUCAAACACCGGUCGCCACCAUG	hdKRed
42	hsa-miR-363-5p	MIMAT0003385	GGUUCCGCGAUCGCGGAUCCAAAUUGCAUCGUGAUCCACCCGAGAUCACACCGGUCGCCACCAUG	hdKRed
43	hsa-miR-145-3p	MIMAT0004601	GGUUCCGCGAUCGCGGAUCCAGAACAGUAUUUCCAGGAAUCCAGAUCACACCGGUCGCCACCAUG	hdKRed
44	hsa-miR-574-5p	MIMAT0004795	GGUUCCGCGAUCGCGGAUCCACACACUCACACACACACACUCAAGAUCCACCGGUCGCCACCAUG	hdKRed
45	hsa-miR-223-3p	MIMAT0000280	GGUUCCGCGAUCGCGGAUCCUGGGGUAUUUGACAAACUGACAAGAUCACACCGGUCGCCACCAUG	hdKRed
46	hsa-miR-4521	MIMAT0019058	GGUUCCGCGAUCGCGGAUCCCUGAGCACAGGACUUCCUUAGCAGAUCACACCGGUCGCCACCAUG	hdKRed
47	hsa-miR-22-5p	MIMAT0004495	GGUUCCGCGAUCGCGGAUCCUAAAGCUUGCCACUGAAGAACUAGAUCACACCGGUCGCCACCAUG	hdKRed
48	hsa-miR-339-3p	MIMAT0004702	GGUUCCGCGAUCGCGGAUCCCGGCUCUGUCGUCGAGGCGCUCAAGAUCCACCGGUCGCCACCAUG	hdKRed
49	hsa-miR-16-2-3p	MIMAT0004518	GGUUCCGCGAUCGCGGAUCCUAAAGCAGCACAGUAAUAUUGGAGAUCACACCGGUCGCCACCAUG	hdKRed
50	hsa-miR-374a-3p	MIMAT0004688	GGUUCCGCGAUCGCGGAUCCAAUUACAAUACAAUCUGAUAAGAGAUCACACCGGUCGCCACCAUG	hdKRed
51	hsa-miR-542-3p	MIMAT0003389	GGUUCCGCGAUCGCGGAUCCUUUCAGUUAUCAAUCUGUCACAAGAUCACACCGGUCGCCACCAUG	hdKRed
52	hsa-miR-452-5p	MIMAT0001635	GGUUCCGCGAUCGCGGAUCCUCAGUUUCCUCUGCAAACAGUUAGAUCACACCGGUCGCCACCAUG	hdKRed
53	hsa-miR-513a-3p	MIMAT0004777	GGUUCCGCGAUCGCGGAUCCCCUUCUCAGAAAGGUGAAAUUUAAGAUCCACCGGUCGCCACCAUG	hdKRed
54	hsa-miR-532-3p	MIMAT0004780	GGUUCCGCGAUCGCGGAUCCUGCAAGCCUUGGGUGUGGGAGGAGAUCACACCGGUCGCCACCAUG	hdKRed
55	hsa-miR-548a-3p	MIMAT0003251	GGUUCCGCGAUCGCGGAUCCGCAAAAGUAAUUGCCAGUUUUGAGAUCACACCGGUCGCCACCAUG	hdKRed
56	hsa-miR-27b-5p	MIMAT0004588	GGUUCCGCGAUCGCGGAUCCGUUCACCAAUCAGCUAAGCUCUAGAUCACACCGGUCGCCACCAUG	hdKRed
57	hsa-miR-3180-5p	MIMAT0015057	GGUUCCGCGAUCGCGGAUCCCGACGUGGGGCGGAGCGUCUGGAGAUCACACCGGUCGCCACCAUG	hdKRed
58	hsa-miR-4324	MIMAT0016876	GGUUCCGCGAUCGCGGAUCCUUAAGGUUAGGGUCUCAGGGAGAUCAAACACCGGUCGCCACCAUG	hdKRed
59	hsa-let-7d-3p	MIMAT0004484	GGUUCCGCGAUCGCGGAUCCAGAAAGGCAGCAGGUCGUAUAGAGAUCACACCGGUCGCCACCAUG	hdKRed
60	hsa-miR-184	MIMAT0000454	GGUUCCGCGAUCGCGGAUCCACCCUUAUCAGUUCUCCGUCCAAGAUCACACCGGUCGCCACCAUG	hdKRed
61	hsa-miR-95	MIMAT0000094	GGUUCCGCGAUCGCGGAUCCUGCUCAAUAAAUACCCGUUGAAAGAUCACACCGGUCGCCACCAUG	hdKRed
62	hsa-miR-664a-5p	MIMAT0005948	GGUUCCGCGAUCGCGGAUCCAUCCAAUCAUUUUCCCUAGCCAGUAGAUCACCGGUCGCCACCAUG	hdKRed
63	hsa-miR-1234-5p	MIMAT0022944	GGUUCCGCGAUCGCGGAUCCCGGCCCCCCCCCCCCCCCCCAGAUCAACACCGGUCGCCACCAUG	hdKRed
64	hsa-miR-185-3p	MIMAT0004611	GGUUCCGCGAUCGCGGAUCCGACCAGAGGAAAGCCAGCCCCUAGAUCACACCGGUCGCCACCAUG	hdKRed
65	hsa-miR-301a-5p	MIMAT0022696	GGUUCCGCGAUCGCGGAUCCAGUAGUGCAAUAAAGUCAGAGCAGAUCACACCGGUCGCCACCAUG	hdKRed
66	hsa-miR-425-3p	MIMAT0001343	GGUUCCGCGAUCGCGGAUCCGGGCGGACACGACAUUCCCGAUAGAUCACACCGGUCGCCACCAUG	hdKRed
67	hsa-miR-497-5p	MIMAT0002820	GGUUCCGCGAUCGCGGAUCCACAAACCACAGUGUGCUGCUGAGAUCAACACCGGUCGCCACCAUG	hdKRed
68	hsa-miR-10b-3p	MIMAT0004556	GGUUCCGCGAUCGCGGAUCCAUUCCCCUAGAAUCGAAUCUGUAGAUCACACCGGUCGCCACCAUG	hdKRed
69	hsa-miR-329	MIMAT0001629	GGUUCCGCGAUCGCGGAUCCAAAGAGGUUAACCAGGUGUGUUAGAUCACACCGGUCGCCACCAUG	hdKRed
70	hsa-miR-144-3p	MIMAT0000436	GGUUCCGCGAUCGCGGAUCCAGUACAUCAUCUAUACUGUAAGAUCAAACACCGGUCGCCACCAUG	hdKRed
71	hsa-miR-211-3p	MIMAT0022694	GGUUCCGCGAUCGCGGAUCCGCACCCCUUUGCUGUCCCUGCAGAUCAACACCGGUCGCCACCAUG	hdKRed
72	hsa-miR-379-5p	MIMAT0000733	GGUUCCGCGAUCGCGGAUCCCCUACGUUCCAUAGUCUACCAAGAUCAACACCGGUCGCCACCAUG	hdKRed
73	hsa-miR-629-5p	MIMAT0004810	GGUUCCGCGAUCGCGGAUCCAGUUCUCCCAACGUAAACCCAAGAUCAACACCGGUCGCCACCAUG	hdKRed
74	hsa-miR-212-5p	MIMAT0022695	GGUUCCGCGAUCGCGGAUCCAGUAAGCAGUCUAGAGCCAAGGUAGAUCCACCGGUCGCCACCAUG	hdKRed

75	hsa-miR-582-5p	MIMAT0003247	GGUUCCGCGAUCGCGGAUCCAGUAACUGGUUGAACAACUGUAAAGAUCCACCGGUCGCCACCAUG	hdKRed
76	hsa-miR-197-5p	MIMAT0022691	GGUUCCGCGAUCGCGGAUCCCCUCCCACUGCCCUCUCUACCCGAGAUCCACCGGUCGCCACCAUG	hdKRed
77	hsa-miR-222-5p	MIMAT0004569	GGUUCCGCGAUCGCGGAUCCAGGAUCUACACUGGCUACUGAGAGAUCACACCGGUCGCCACCAUG	hdKRed
78	hsa-miR-93-3p	MIMAT0004509	GGUUCCGCGAUCGCGGAUCCCGGGAAGUGCUAGCUCAGCAGUAGAUCACACCGGUCGCCACCAUG	hdKRed
79	hsa-miR-483-5p	MIMAT0004761	GGUUCCGCGAUCGCGGAUCCCUCCCUUCUUUCCUCCCGUCUUAGAUCACACCGGUCGCCACCAUG	hdKRed
80	hsa-miR-576-5p	MIMAT0003241	GGUUCCGCGAUCGCGGAUCCAAAGACGUGGAGAAAUUAGAAUAGAUCACACCGGUCGCCACCAUG	hdKRed
81	hsa-miR-146a-3p	MIMAT0004608	GGUUCCGCGAUCGCGGAUCCCUGAAGAACUGAAUUUCAGAGGAGAUCACACCGGUCGCCACCAUG	hdKRed
82	hsa-miR-584-3p	MIMAT0022708	GGUUCCGCGAUCGCGGAUCCAGCCUGGUUGGCCUGGAACUGAAGAUCACACCGGUCGCCACCAUG	hdKRed
83	hsa-miR-34a-3p	MIMAT0004557	GGUUCCGCGAUCGCGGAUCCAGGGCAGUAUACUUGCUGAUUGAGAUCACACCGGUCGCCACCAUG	hdKRed
84	hsa-miR-190a	MIMAT0000458	GGUUCCGCGAUCGCGGAUCCACCUAAUAUAUCAAACAUAUCAAGAUCACACCGGUCGCCACCAUG	hdKRed
85	hsa-miR-383	MIMAT0000738	GGUUCCGCGAUCGCGGAUCCAGCCACAAUCACCUUCUGAUCUAGAUCACACCGGUCGCCACCAUG	hdKRed
86	hsa-miR-326	MIMAT0000756	GGUUCCGCGAUCGCGGAUCCCUGGAGGAAGGGCCCAGAGGAGAUCAAACACCGGUCGCCACCAUG	hdKRed
87	hsa-miR-576-3p	MIMAT0004796	GGUUCCGCGAUCGCGGAUCCGAUUCCAAUUUUUCCACAUCUUAGAUCACACCGGUCGCCACCAUG	hdKRed
88	hsa-miR-219-5p	MIMAT0000276	GGUUCCGCGAUCGCGGAUCCAGAAUUGCGUUUGGACAAUCAAGAUCAACACCGGUCGCCACCAUG	hdKRed
89	hsa-miR-342-5p	MIMAT0004694	GGUUCCGCGAUCGCGGAUCCUCAAUCACAGAUAGCACCCCUAGAUCAACACCGGUCGCCACCAUG	hdKRed
90	hsa-miR-590-3p	MIMAT0004801	GGUUCCGCGAUCGCGGAUCCACUAGCUUAUACAUAAAAUUAAGAUCAACACCGGUCGCCACCAUG	hdKRed
91	hsa-miR-137	MIMAT0000429	GGUUCCGCGAUCGCGGAUCCCUACGCGUAUUCUUAAGCAAUAAAGAUCCACCGGUCGCCACCAUG	hdKRed
92	hsa-miR-298	MIMAT0004901	GGUUCCGCGAUCGCGGAUCCUGGGAGAACCUCCCUGCUUCUGCUAGAUCACCGGUCGCCACCAUG	hdKRed
93	hsa-miR-325	MIMAT0000771	GGUUCCGCGAUCGCGGAUCCACACUUACUGGACACCUACUAGGAGAUCCACCGGUCGCCACCAUG	hdKRed
94	hsa-miR-449a	MIMAT0001541	GGUUCCGCGAUCGCGGAUCCACCAGCUAACAAUACACUGCCAAGAUCACACCGGUCGCCACCAUG	hdKRed
95	hsa-miR-498	MIMAT0002824	GGUUCCGCGAUCGCGGAUCCGAAAAACGCCCCCUGGCUUGAAAAGAUCCACCGGUCGCCACCAUG	hdKRed
96	hsa-miR-661	MIMAT0003324	GGUUCCGCGAUCGCGGAUCCACGCGCAGGCCAGAGACCCAGGCAAGAUCACCGGUCGCCACCAUG	hdKRed

*[1] The first G is the transcription start site where an anti-reverse cap analog is incorporated. The last AUG is the start codon of the reporter fluorescent protein.

*[2] Oligo-DNAs that start with T7 promoter (5’-CGACTCACTATA) followed by the DNA sequence of 5’ UTR listed in this table were used for the amplification of IVT templates via fusion PCR.

Table S3. List of primers, single-stranded oligo DNAs, and plasmids for PCR amplification.

Type	Forward Primer	Sequence	Reverse Primer	Sequence
**hmAG1 ORF**	FwdhmAG1	CACCGGTCGCCACCATGGTGAGCGTGATCAAGCCCG	RevhmAG1	GCCCCGCAGAAGGTCTAGATTCACTTGGCCTGGCTGGGC
**hmKO2 ORF**	FwdhmKO2	CACCGGTCGCCACCATGGTGAGTGTGATTAAACCAG AGATG	RevhmKO2	GCCCCGCAGAAGGTCTAGATTCAGGAATGAGCTACTGCAT CTTCTACCTG
**tagBFP ORF**	FwdtagBFP	CACCGGTCGCCACCATGGGATCCAGCGAG	RevtagBFP	GCCCCGCAGAAGGTCTAGACTATCACTCGAGATGCATATG AGATC
**hdKRed ORF**	FwdhdKRed	CACCGGTCGCCACCATGGTGAGCGTGATCGCCAAG	RevhdKRed	GCCCCGCAGAAGGTCTAGATTCAGCCCAGCAGGCTGTGC
**Control 5' UTR**	Fwd5UTR	CAGTGAATTGTAATACGACTCACTATAG	Rev5UTR	CAGTGAATTGTAATACGACTCACTATAG
**3' UTR**	Fwd3UTR	TCTAGACCTTCTGCGGGGC	Rev3UTR	TTTTTTTTTTTTTTTTTTTTCCTACTCAGGCTTTATTCAA AGACCAAG
**IVT template (fusion PCR)**	T7Fwd5UTR	CAGTGAATTGTAATACGACTCACTATAG	Rev120A	TTTTTTTTTTTTTTTTTTTTTTTTTTTTTTTTTTTTTTTT TTTTTTTTTTTTTTTTTTTTTTTTTTTTTTTTTTTTTTTT TTTTTTTTTTTTTTTTTTTTTTTTTTTTTTTTTTTTTTTT CCTACTCAGGCTTTATTCA

Template DNA	Sequence
temp5UTR	CAGTGAATTGTAATACGACTCACTATAGGGCGAATTAAGAGAGAAAAGAAGAGTAAGAAGAAATATAAGACACCGGTCGCCACCATG
temp3UTR	TCTAGACCTTCTGCGGGGCTTGCCTTCTGGCCATGCCCTTCTTCTCTCCCTTGCACCTGTACCTCTTGGTCTTTGAATAAAGCCTGAGTAGG
**Template DNA**	**Plasmids**
hmAG1	pFucci-S/G2/M Green (Amalgaam)
hmKO2	pFucci-G1 Orange (Amalgaam)
tagBFP	pSRT-tagBFP (ref. *20*) derived from pTagBFP-Tubulin (Evrogen)
hdKRed	pNP-hdKeima-Red (Amalgaam)

Table S4. List of experimental conditions.

Experiment	Item	Cell	Transfected RNA	Protocol
Five-slot mRNAs to two different miRNAs	Fig. 2B,C<br>Table S1	HeLa (50,000 cells)	hmAG1 (five-slot reporter, 80 ng)	Forward transfection
			tagBFP (control, 100 ng)	
			miRNA inhibitor (total 4 pmol, 2 pmol x2)	
Five-slot mRNAs to three different miRNAs	Fig. 2C<br>Table S1	HeLa (50,000 cells)	hmAG1 (five-slot reporter, 80 ng)	Forward transfection
			tagBFP (control, 100 ng)	
			miRNA inhibitor (total 6 pmol, 2 pmol x3)	
Five-slot mRNAs with multiple target sites to one miRNA	Fig. 3B,C, S2B<br>Table S1	HeLa (50,000 cells)	hmAG1 (five/single-slot reporter, 80 ng)	Forward transfection
			tagBFP (control, 100 ng)	
			miRNA inhibitor (2 pmol)	
Screening of miRNA activity profiles*[1]	Fig. 4A–C, 5A–C<br>Table S2	All cells used in this study (50,000 – 100,000 cells)	hmAG1 (single-slot reporter, 40 ng)	Reverse transfection
			tagBFP (single-slot reporter, 90 ng)	
			hdKRed (single-slot reporter, 400 ng)	
			hmKO2 (control, 20 ng)	
	Fig. 5A–C<br>Table S2	hiPSCs*[2] *[3]	hmAG1 (single-slot reporter, 40 ng)	Forward*[2] transfection
			tagBFP (single-slot reporter, 90 ng)	
			hdKRed (single-slot reporter, 400 ng)	
			hmKO2 (control, 20 ng)	
2D density plot	Fig. 4G, S4<br>Table S1	All cells used in this study	hmAG1 (40 ng)	Reverse transfection
			tagBFP (90 ng)	
			hdKRed (400 ng)	
			hmKO2 (20 ng)	
	Fig. 5G, S5<br>Table S1	hiPSCs*[2] *[3]	hmAG1 (40 ng)	Forward*[2] transfection
			tagBFP (90 ng)	
			hdKRed (400 ng)	
			hmKO2 (20 ng)	

*[1] We used the same lot of four-mRNA mixtures throughout the screening to avoid noises in miRNA activity detection caused by mRNA mixing.

*[2] hiPSCs were cultured without bFGF for 1-9 days on 24-well plates and subjected to forward transfection.

*[3] Transfections into 50,000 hiPSCs on days 0 and 14 were performed with the reverse transfection protocol.
